# Global estimates of expected and preventable cervical cancers among girls born between 2005 and 2014: a birth cohort analysis

**DOI:** 10.1016/S2468-2667(21)00046-3

**Published:** 2021-04-15

**Authors:** Maxime Bonjour, Hadrien Charvat, Eduardo L Franco, Marion Piñeros, Gary M Clifford, Freddie Bray, Iacopo Baussano

**Affiliations:** a Early Detection, Prevention and Infections Branch, International Agency for Research on Cancer (IARC/WHO), Lyon, France; b Cancer Surveillance Branch, International Agency for Research on Cancer (IARC/WHO), Lyon, France; c Université de Lyon, Université Lyon 1, Lyon, France; d Division of Cancer Epidemiology, McGill University, Montreal, QC, Canada

## Abstract

**Background:**

WHO has launched an initiative aiming to eliminate cervical cancer as a public health problem. Elimination is a long-term target that needs long-lasting commitment. To support local authorities in implementing human papillomavirus (HPV) vaccination, we provide regional and country-specific estimates of cervical cancer burden and the projected impact of HPV vaccination among today's young girls who could develop cervical cancer if not vaccinated.

**Methods:**

The expected number of cervical cancer cases in the absence of vaccination among girls born between 2005 and 2014 was quantified by combining age-specific incidence rates from GLOBOCAN 2018 and cohort-specific mortality rates by age from UN demographic projections. Preventable cancers were estimated on the basis of HPV prevalence reduction attributable to vaccination and the relative contribution of each HPV type to cervical cancer incidence. We assessed the number of cervical cancer cases preventable through vaccines targeting HPV types 16 and 18, with and without cross-protection, and through vaccines targeting HPV types 16, 18, 31, 33, 45, 52, and 58.

**Findings:**

Globally, without vaccination, the burden of cervical cancer in these birth cohorts is expected to reach 11·6 million (95% uncertainty interval 11·4–12·0) cases by 2094. Approximately 75% of the burden will be concentrated in 25 countries mostly located in Africa and Asia, where the future number of cases is expected to increase manyfold, reaching 5·6 million (5·4–6·0) cases in Africa and 4·5 million (4·4–4·6) cases in Asia. Worldwide immunisation with an HPV vaccine targeted to HPV types 16 and 18, with cross-protection against HPV types 31, 33, and 45, could prevent about 8·7 million (8·5–9·0) cases.

**Interpretation:**

Detailed estimates of the increasing burden of cervical cancer and projected impact of HPV vaccination is of immediate relevance to public health decision makers. Shifting the focus of projections towards recently born girls who could develop cervical cancer if not vaccinated is fundamental to overcome stakeholders' hesitancy towards HPV vaccination.

**Funding:**

Bill & Melinda Gates Foundation, Canadian Institutes of Health Research.

## Introduction

Approximately 570 000 new cases and 311 000 deaths from cervical cancer occurred in 2018. Cervical cancer is the fourth most common cancer in women worldwide and the greatest contributor to cancer-related mortality among women in sub-Saharan Africa.^1^ There remain marked variations in incidence rates internationally, ranging from fewer than two cases per 100 000 women to 85 cases per 100 000 women for the period 2008–12.^1^

Infection with high-risk (ie, carcinogenic) types of human papillomavirus (HPV) is a necessary cause of cervical cancer.^2^ Effective prophylactic vaccines against the high-risk HPV types most frequently found in cervical cancer have been available for more than 10 years^3^ and have shown high safety^4^ and efficacy against persistent HPV infections, precancerous lesions^3^ and invasive cervical cancers,^5^ while HPV vaccination programmes have been shown to be cost-effective in a wide range of settings worldwide.^6^ However, local stakeholders face multiple obstacles to implementing and scaling up HPV vaccination as a component of their national expanded programmes of immunisation. These obstacles include vaccine supply shortage,^7^ budgetary constraints,^8^ vaccine hesitancy,^9^ and massive disruption of health-care services such as those occurring during the COVID-19 pandemic. As a result, by mid-2019, only 96 countries, mostly high income, had introduced the vaccine. Moreover, the reported coverage of target birth cohorts is currently unequal throughout the world and, for many countries, often considerably lower than the recommended threshold of 90%.^10^

The availability of effective prophylactic HPV vaccines, of validated HPV assays, and of effective treatment of cervical precancerous lesions and cervical cancer underpins the call for global action towards the elimination of cervical cancer as a public health problem launched by WHO in November, 2020.^11^ The overarching target of this initiative is to reduce the age-standardised incidence rate of cervical cancer to fewer than four cases per 100 000 women worldwide by vaccinating 90% of all girls by age 15 years, screening 70% of women twice in the age range of 35–45 years, and treating at least 90% of women diagnosed with cervical disease (including 90% women with cervical precancer and 90% women with invasive malignancy).

**Research in context**

**Evidence before this study**

WHO has launched a global initiative aiming to eliminate cervical cancer as a public health problem. Scaling up in all countries of vaccination, screening, and precancer treatment, in addition to invasive cancer treatment and palliative care, underpins the global elimination strategy. We searched PubMed for studies published from Jan 1, 2000, to Nov 30, 2020, with the search terms “cervical cancer” or “human papillomavirus” or “HPV” and “elimination” and “model” or “modelling”. Four studies were retained out of 226 identified references. Findings from these studies suggest that widespread coverage of both human papillomavirus (HPV) vaccination and cervical screening has the potential to avert about 13 million cervical cancer cases by 2069. The reported findings also suggest that cervical cancer elimination might be attainable within a few decades in high-income countries but will take longer in low-income and middle-income countries (LMICs), and will be achieved towards the end of the 21st century in a number of sub-Saharan African countries where rates are currently the highest worldwide. However, by mid-2019, only 96 countries, mostly high income, had introduced HPV vaccination. The reported coverage of target birth cohorts is currently unequal throughout the world and, for many countries, often considerably lower than the recommended threshold of 90%. Therefore, worldwide efforts to bolster implementation and scale-up of vaccination programmes are essential, particularly considering that HPV vaccination is highly cost-effective, as recently confirmed by an updated global assessment done with the Papillomavirus Rapid Interface for Modelling and Economics.

**Added value of this study**

At a local level, public health decision makers need predictions targeted to today's girls and young women at an eligible age for HPV vaccination to inform and frame their vaccination plans. This analysis assesses the expected burden of cervical cancer in the absence of HPV vaccination and the impact of vaccination from the perspective of women born between 2005 and 2014, who could expect to be protected against cervical cancer during their lifespan. We have focused our attention on cohort-specific cervical cancer burden as it is an indicator of direct relevance to decision makers wanting to assess the risk to today's young girls at an eligible age for HPV vaccination. Public health authorities, particularly in LMICs, are facing multiple obstacles, such as vaccine supply shortage, budgetary constraints, and vaccine hesitancy, to implementing and scaling up HPV vaccination as a component of their national expanded programme of immunisation. Our regional and country-specific estimates could provide key stakeholders with clear information to reinforce commitment and overcome reluctance towards HPV vaccination.

**Implications of all the available evidence**

Recent modelling exercises have shown that global cervical cancer elimination is a long-term target that will be achieved by scaling up vaccination, screening, and treatment of precancers and invasive cancers. Therefore, at a local level, to aim at elimination, it is essential to ensure long-lasting commitment to the allocation of adequate resources. Durable political and financial commitment towards HPV vaccination is effectively supported by the direct quantification of its impact on cervical cancer burden among today's girls and young women.

Mathematical modelling exercises suggest that this goal might be attainable within a few decades in high-income countries, but will take longer in low-income and middle-income countries (LMICs), and might only be reached towards the end of this century in a number of sub-Saharan African countries where rates are currently the highest worldwide.^12^, ^13^, ^14^ Focusing on the potential impact of HPV vaccination on today's young girls who are at risk of developing cervical cancer if not vaccinated is crucial to supporting the long-term commitments of public health authorities to implementing HPV vaccination.^15^ Since HPV vaccination programmes are introduced in the context of competing needs for finite resources, local stakeholders and decision makers need to envisage the expected burden of cervical cancer in the local population and potential impact of HPV vaccination among girls and young women eligible for vaccination. To address this need, in this Article we quantify the future burden of cervical cancer among a generation of women born between 2005 and 2014 who are, or will be, eligible for HPV vaccination, and estimate the number of cancers that would be preventable through vaccination in the same birth cohorts, presenting the results for 185 countries worldwide.

## Methods

### Expected lifetime number of cervical cancers

We estimated the expected lifetime number of cervical cancers among birth cohorts aged 15 years between 2020 and 2029 (ie, born between 2005 and 2014) in the absence of vaccination, along with the corresponding 95% uncertainty intervals (UIs), by combining age-specific incidence projections from GLOBOCAN and the cohort-specific mortality rates by age from the UN Department of Economic and Social Affairs Population Division, allowing for the competing risk of dying from any cause before being diagnosed with cervical cancer (see appendix pp 2–4 for technical details). Accounting for cohort-specific mortality rates by age enabled us to incorporate future demographic changes in our projections. The GLOBOCAN 2018 database, as provided by the International Agency for Research on Cancer and presented in the Global Cancer Observatory, includes national estimates of the number of new cervical cancer cases in 185 countries in 2018.^16^, ^17^ To assess the effect of future demographic changes on country-specific cervical cancer burden, we calculated the ratio of future-to-current number of cervical cancers cases, comparing the average number of expected cases across birth cohorts born between 2005 and 2014 in the absence of vaccination versus the total number of cases estimated in 2018. For the sake of simplicity, we assumed no change in annual incidence of cervical cancer attributable to modifications in either sexual behaviour or cervical cancer screening practices.

### Number of preventable cervical cancers

The number of cervical cancer cases that would be preventable through vaccination between ages 15 and 79 years that would be reached between the years 2085 and 2094 in each country was then calculated by reducing the age-specific incidence of cervical cancer proportionally according to the relative contribution of each high-risk HPV type to cervical cancer incidence and assuming the elimination of HPV types targeted by the vaccine—ie, the highest HPV prevalence reduction achievable through a vaccination programme. The number of cervical cancer cases prevented in each country with suboptimal levels of HPV prevalence reduction attributable to vaccination was also estimated by ranging the prevalence reduction from 60% to 100% in all countries. Similar levels of prevalence reduction can result from different combinations of direct and indirect effects of specific characteristics of a vaccination programme (such as type of vaccine adopted, immunisation schedule and coverage, and targeted sex and age groups), as well as population-specific sexual behaviour.^18^, ^19^ Here, we provide estimates of the number of preventable cancers through HPV vaccination irrespective of the combination of specific vaccination scenarios.

As licensed HPV vaccines do not target all high-risk HPV types, the expected reduction of cervical cancer incidence attributable to vaccination in a given population is dependent on the distribution of HPV types in that population. The relative contributions of HPV types 16 and 18; types 31, 33, 45, 52, and 58; and other high-risk HPV types to the cervical cancer burden were derived from a published meta-analysis providing supra-national regional estimates. For each country, we used the corresponding regional estimate.^20^ To characterise India more accurately, we used country-specific estimates.^21^, ^22^ We assessed the number of cervical cancers preventable through vaccines targeting HPV types 16 and 18, with and without cross-protection, and of vaccines targeting HPV types 16, 18, 31, 33, 45, 52, and 58. For vaccines targeting HPV types 16 and 18, cross-protection was modelled assuming a 50% prevalence reduction of HPV types 31, 33, and 45 attributable to vaccination.^23^ Vaccine-induced immune protection was assumed to be lifelong.

### Country classification

We present our results for the 185 countries included in GLOBOCAN by continent, according to their contribution to the expected absolute burden of cervical cancer among women born between 2005 and 2014 (hereafter referred to as burden) and grouped by their human development index (HDI) in the year 2018, as reported by the UN Development Programme. HDI is a summary measure of average achievement in key dimensions of human development in a country (long and healthy life, knowledge, and standard of living). We assessed the correlation between the ratio of future-to-current number of cervical cancers cases and country-specific HDI using the Pearson's coefficient (*r*). To classify countries according to their contribution to the overall cervical cancer burden, countries were sorted by the expected number of cases and subsequently grouped into the following five categories: very high burden (countries accounting for up to 50% of all cervical cancer cases worldwide), high burden (countries accounting for the next 25% of all cases), medium burden (countries accounting for the next 15% of all cases), low burden (countries accounting for the next 9% of all cases), and very low burden (countries accounting for the final 1% of all cases). HDI was stratified into low–middle HDI (<0·70), high HDI (0·70–0·79), and very high HDI (≥0·80). All analyses were done with R (version 3.6.1).

### Role of the funding source

The funder of the study had no role in study design, data collection, data analysis, data interpretation, or writing of the report.

## Results

**Figure 1:**
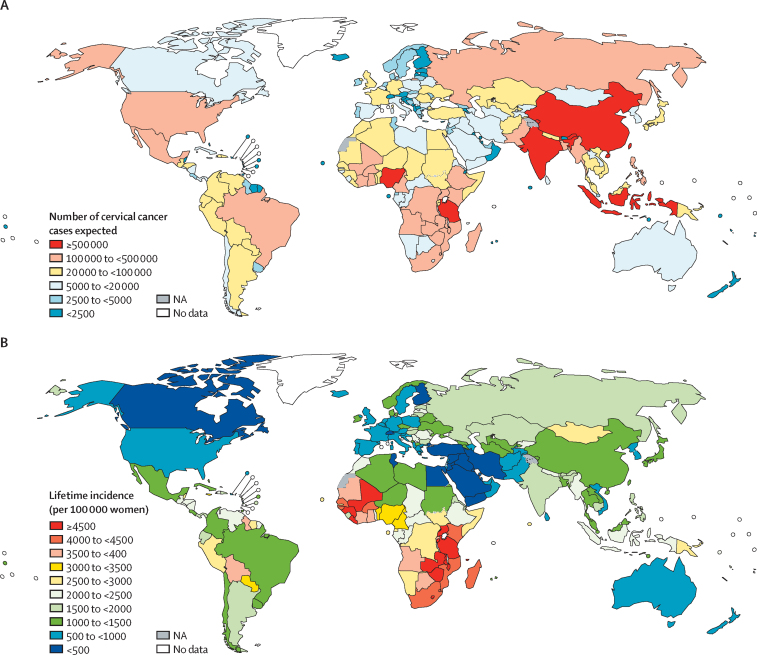
Number of cervical cancer cases (A) and lifetime incidence (B) expected among women born between 2005 and 2014, by country NA=not applicable.

Globally, in the absence of vaccination, we would expect 11·6 million (95% UI 11·4–12·0) cervical cancer cases to occur among women born between 2005 and 2014 (table 1). Africa and Asia are expected to share the largest burden of cervical cancer cases, with more than 80% of all cases (table 1; figure 1A). Some 0·9 million (0·8–0·9) cases are expected to occur in Latin America, whereas 0·6 million (0·6–0·6) cases are expected in North America, Europe, and Oceania (table 1; figure 1A). The large size of the population in some countries is a key determinant of cervical cancer burden, as shown when considering expected lifetime incidence (per 100 000 women) among women born between 2005 and 2014, by country (figure 1B).

**Table 1:** Number of women at risk and number of cervical cancer cases expected among women born between 2005 and 2014 by continent, cervical cancer burden category, and 2018 human development index

	**Number of women at risk**	**Cases expected in the absence of vaccination**	
		Number (95% uncertainty interval)	Percentage of total cases in each category
**Continent**			
Africa	165 606 523	5 648 149 (5 428 370–6 021 112)	48·7%
Asia	344 978 554	4 486 109 (4 372 716–4 643 003)	38·7%
Europe	38 508 937	416 241 (410 384–423 343)	3·6%
Latin America	52 222 051	863 532 (835 639–919 393)	7·4%
North America	22 124 133	140 961 (137 550–144 461)	1·2%
Oceania	3 061 127	42 855 (39 073–47 384)	0·4%
Cervical cancer burden*			
Very high	292 719 493	5 949 749 (5 745 857–6 186 696)	51·3%
High	136 428 165	2 808 840 (2 671 891–3 045 844)	24·2%
Medium	77 561 473	1 697 817 (1 597 925–1 890 372)	14·6%
Low	106 942 955	1 027 948 (992 516–1 118 556)	8·9%
Very low	12 849 239	113 492 (109 602–120 963)	1·0%
**Human development index**†			
Low–middle	352 464 260	8 025 880 (7 794 459–8 447 380)	69·2%
High	186 108 791	2 775 193 (2 720 782–2 837 271)	23·9%
Very high	87 928 274	796 774 (786 593–810 166)	6·9%
Total	626 501 325	11 597 847 (11 366 107–12 027 739)	100·0%

* Individual countries were sorted according to the expected number of cervical cancer cases, then grouped into the following categories: very high burden (eight countries accounting for up to 50% of all cases worldwide), high burden (17 countries accounting for the next 25% of all cases), medium burden (25 countries accounting for the next 15%), low burden (68 countries accounting for the next 9%), and very low burden (67 countries accounting for the remaining 1%).

† Low–middle: <0·70; high: 0·70–0·79; and very high: ≥0·80.

**Figure 2:**
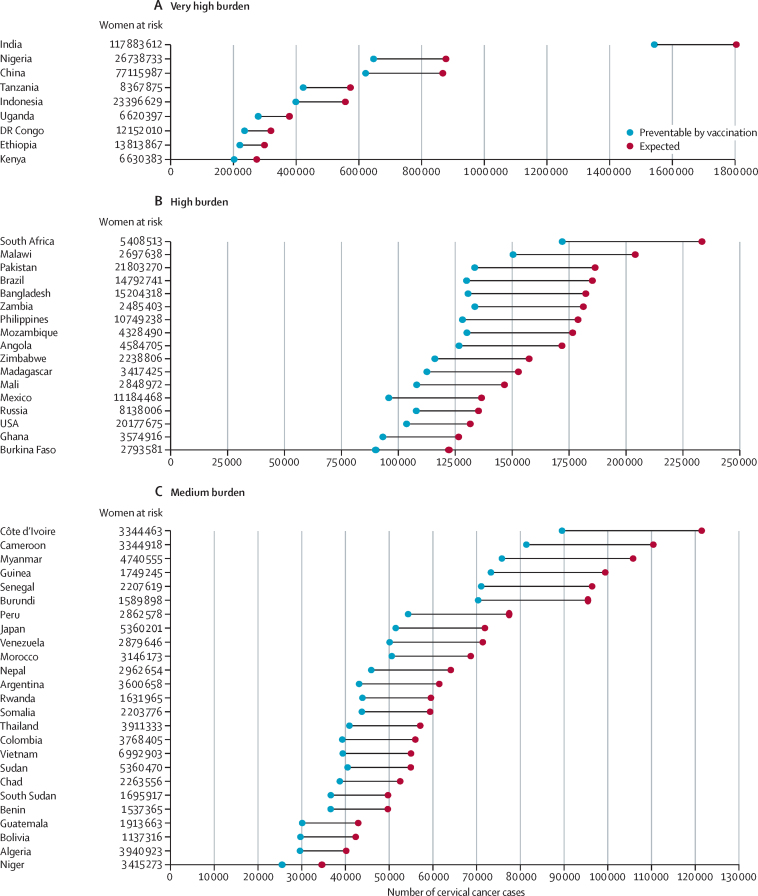

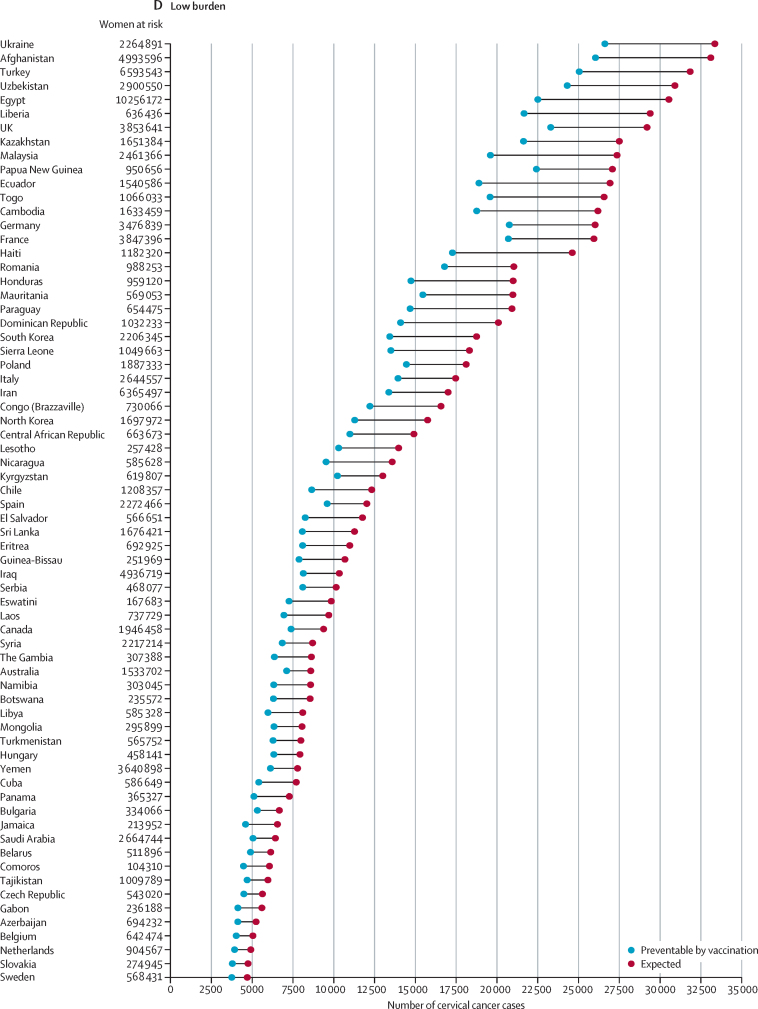

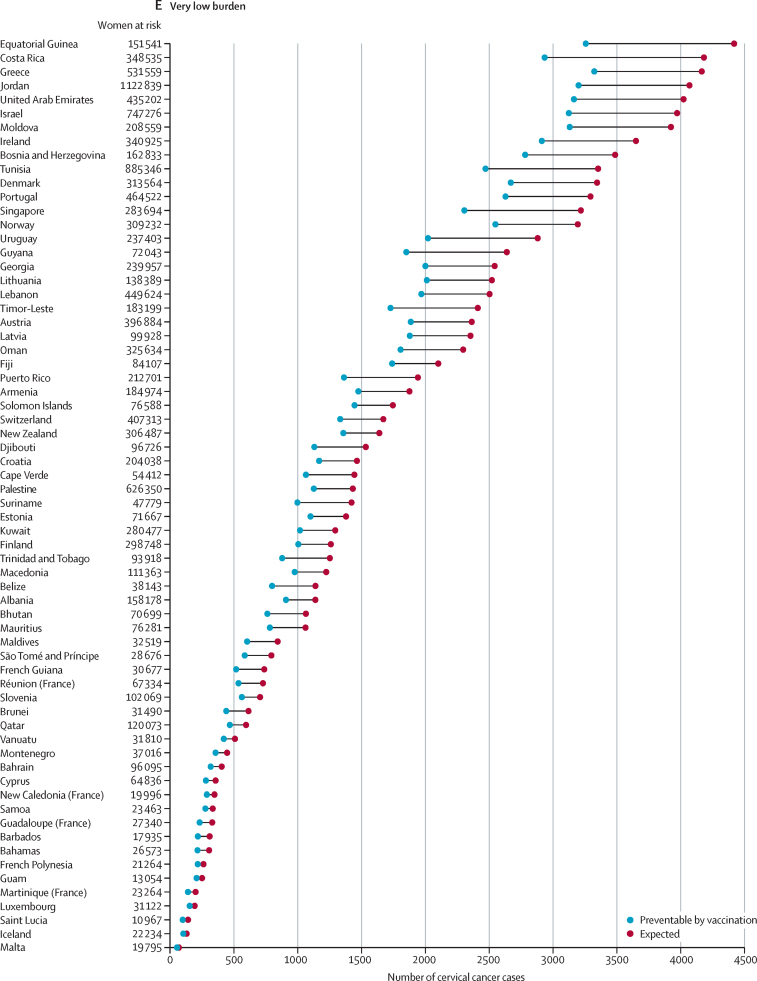
Country-specific numbers of cervical cancer cases expected among women born between 2005 and 2014 in the absence of vaccination and preventable through vaccination programmes Countries are grouped according to their contribution to the overall cervical cancer burden, with countries sorted according to the expected number of cervical cancer cases and subsequently grouped into the following five categories: very high burden (accounting for up to 50% of all cancer cases worldwide), high burden (accounting for the next 25% of all cases), medium burden (accounting for the next 15%), low burden (accounting for the next 9%), and very low burden (accounting for the remaining 1%).

When countries are grouped according to the expected burden of cervical cancer in the absence of vaccination, 51·3% of the overall expected burden (5·9 million [95% UI 5·7–6·2] cervical cancer cases over a lifetime) would affect birth cohorts in India, Nigeria, China, Tanzania, Indonesia, Uganda, Democratic Republic of the Congo, Ethiopia, and Kenya (figure 2A). Another 2·8 million (2·7–3·0) cases, corresponding to 24·2%, would be concentrated in 17 countries mostly in sub-Saharan Africa (South Africa, Malawi, Zambia, Mozambique, Angola, Zimbabwe, Madagascar, Mali, Ghana, and Burkina Faso), Asia (Pakistan, Bangladesh, and the Philippines), the Americas (Brazil, Mexico, and the USA), and in Russia (figure 2B). The residual 24·5% (2·8 million [2·7–3·1] cases) of the overall projected cervical cancer burden in unvaccinated birth cohorts is expected to occur in the remaining 159 countries (figure 2C–E). Overall, the estimated burden of cervical cancer decreases according to HDI level, from 8·0 million (7·8–8·4) cases in countries with low–middle HDI to 0·8 million (0·8–0·8) cases in countries with very high HDI (table 1).

**Figure 3:**
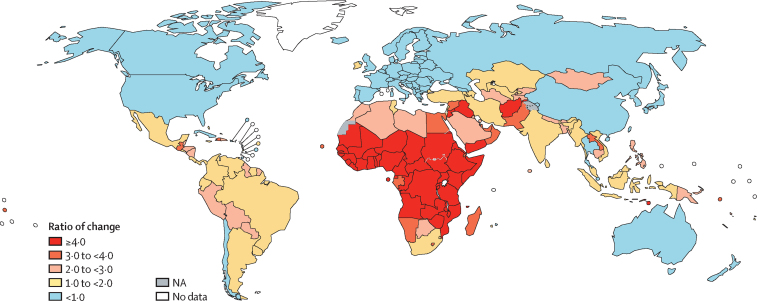
Ratio of the average number of expected cervical cancer cases across birth cohorts born between 2005 and 2014 in the absence of vaccination versus the total number of cases estimated in 2018 NA=not applicable.

To illustrate the effect of future demographic changes on country-specific cervical cancer burden, we compared the average number of expected cervical cancer cases across birth cohorts born between 2005 and 2014 in the absence of vaccination to the number of cases estimated by GLOBOCAN for the year 2018 (figure 3). Demographic changes are expected to increase the cervical cancer burden, particularly in countries that already have high or very high burden. In most of sub-Saharan Africa, the future burden could be more than four times higher than in 2018, whereas in parts of Asia, as well as in parts of Latin America, it is expected to be between two and three times higher. Conversely, a relative reduction in the absolute number of cancers is expected in China, Russia, and most countries with high or very high HDI. Overall, a strong negative correlation (*r* = –0·5) was observed between the ratio of future-to-current number of cervical cancers cases and country-specific HDI (appendix p 32).

**Figure 4:**
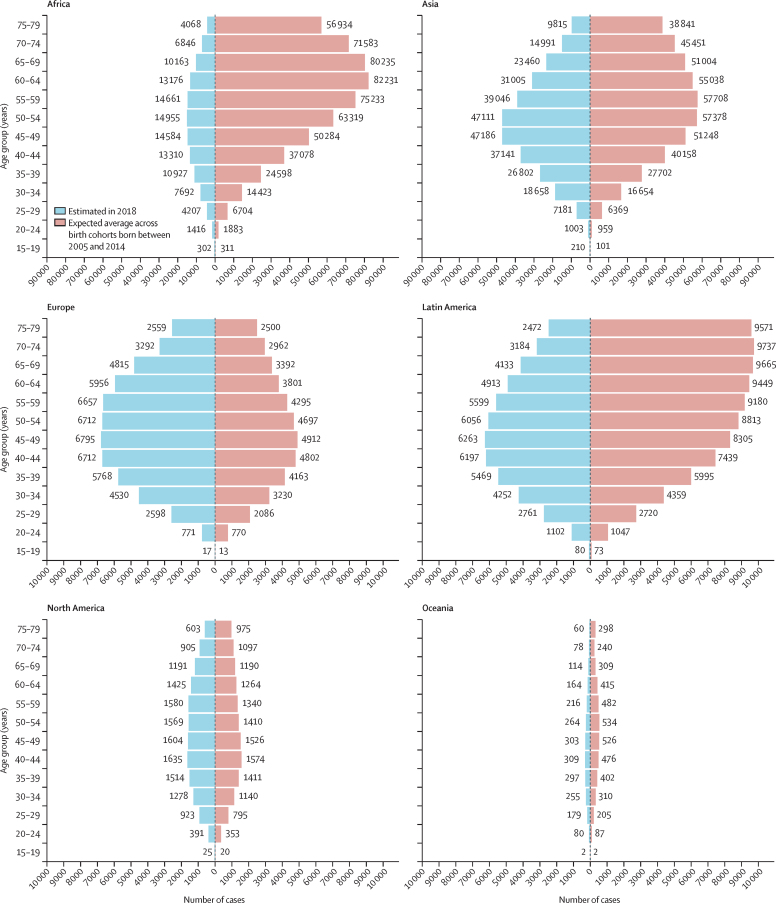
Age-specific distribution of average number of expected cervical cancer cases across birth cohorts born between 2005 and 2014 in the absence of vaccination, compared with those estimated in 2018, by continent

Demographic changes are also expected to affect the future age distribution of cervical cancers. In 2018, 72·7% of all cases worldwide occurred before 60 years of age, with a similar age distribution of cases across continents (figure 4). By contrast, 53·6% (6·2 million [6·1–6·4] cases) of all future cervical cancers expected in the absence of vaccination among women born between 2005 and 2014 will occur by 60 years of age (figure 4). Importantly, the expected age distribution of cancer cases is expected to differ by continent. In Africa, Asia, and Latin America approximately 50% are expected to occur before 60 years of age, whereas in Europe, North America, and Oceania approximately 70% will occur before 60 years of age.

Worldwide immunisation with an HPV vaccine targeted to HPV types 16 and 18, with cross-protection against HPV types 31, 33, and 45 through vaccination programmes with the highest effectiveness could prevent about 8·7 million (8·5–9·0) cervical cancer cases (ie, 75·3% of all expected cancers) among women born between 2005 and 2014 (table 2). The largest number of cases would be averted in Africa and Asia (table 2). In particular, 4·6 million (4·4–4·8) cases—corresponding to 52·3% of all preventable cases worldwide—are potentially avertable in very high burden countries, 2·1 million (2·0–2·2) cancers (23·6% of all preventable cases worldwide) in high burden countries, and 2·1 million (2·0–2·3) cancers (24% of all preventable cases worldwide) in countries with medium to very low burden (figure 2; table 2). More than 6·1 million (5·9–6·4) cervical cancer cases are preventable through vaccination in countries with low–middle HDI (table 2).

**Table 2:** Preventable cervical cancer cases among women born between 2005 and 2014 by continent, cervical cancer burden category, and 2018 human development index

	**Number of preventable cases**	**Percentage of cases preventable through vaccination in each category**
**Continent**		
Africa	4 162 782 (4 000 569–4 437 821)	73·7%
Asia	3 480 802 (3 380 678–3 608 856)	77·6%
Europe	332 124 (327 352–337 944)	79·8%
Latin America	605 918 (586 145–644 957)	70·2%
North America	111 009 (107 869–114 330)	78·8%
Oceania	35 485 (32 318–39 271)	82·8%
Cervical cancer burden*		
Very high	4 568 726 (4 405 526–4 755 927)	76·8%
High	2 062 358 (1 961 833–2 236 491)	73·4%
Medium	1 231 586 (1 158 349–1 372 443)	72·5%
Low	778 124 (752 326–844 285)	75·7%
Very low	87 326 (84 360–93 058)	76·9%
**Human development index**†		
Low–middle	6 117 421 (5 939 136–6 430 904)	76·2%
High	1 994 697 (1 954 640–2 039 514)	71·9%
Very high	616 002 (607 835–626 440)	77·3%
Total	8 728 120 (8 549 700–9 049 217)	75·3%

* Individual countries were sorted according to the expected number of cervical cancer cases, then grouped into the following categories: very high burden (eight countries accounting for up to 50% of all cancer cases worldwide), high burden (17 countries accounting for the next 25% of all cases), medium burden (25 countries accounting for the next 15%), low burden (68 countries accounting for the next 9%), and very low burden (67 countries accounting for the remaining 1%).

† Low–middle: <0·70; high: 0·70–0·79; and very high: ≥0·80.

In the appendix, we report detailed country-specific estimates, with 95% UIs, of the number of lifetime cervical cancer cases expected in the absence of HPV vaccination and potentially avoidable through vaccination at differing levels of vaccine effectiveness in the population (appendix pp 5–13). We also report the expected impact of vaccines targeting HPV types 16 and 18 without cross-protection, which are expected to prevent 8·0 million (7·8–8·3) cervical cancer cases (appendix pp 14–22), and of vaccines targeting HPV types 16, 18, 31, 33, 45, 52, and 58 (appendix pp 23–31), which are expected to prevent 10·2 million (10·0–10·6) cases. Finally, we have also estimated the expected impact of these vaccination programmes with suboptimal levels of reduction of HPV prevalence attributable to vaccination (appendix pp 5–31). Cervical cancer cases prevented globally could range from 5·2 million at 60% HPV infections averted through vaccination to 8·7 million at 100% vaccination effectiveness across birth cohorts born between 2005 and 2014.

## Discussion

Globally, in the absence of vaccination, 11·6 million cervical cancer cases are expected in all women born between 2005 and 2014 who are, or will be, eligible for HPV vaccination. In these birth cohorts, HPV vaccination (with a vaccine targeted against HPV types 16 and 18, and assuming partial cross-protection against HPV types 31, 33, and 45) could prevent 8·7 million cases. 95% of all cases preventable through vaccination would be averted in Africa, Asia, and Latin America, 76% in 26 countries with an expected very high or high burden, and 70% in countries with low–middle HDI.

Previous publications suggest that widespread coverage of both HPV vaccination and cervical cancer screening has the potential to avert about 13 million cervical cancer cases by 2069 worldwide.^14^ Simulation exercises focused on LMICs suggest that successful implementation of the cervical cancer elimination strategy by 2030 would reduce cervical cancer incidence to 0·7 per 100 000 women^12^ and mortality to 0·2 per 100 000 women by 2120.^13^ Furthermore, HPV vaccination is predicted to be cost-effective.^15^, ^24^ However, many LMICs will not reach the cervical cancer elimination threshold until towards the end of the 21st century.^13^ To complement findings from previous modelling studies,^12^, ^13^, ^14^ the focus here has been to show that the burden of cervical cancer and projected impact of HPV vaccination in a population can be quantified among today's girls and young women. We believe this novel way of presenting the impact of the HPV vaccine on specific generations of girls could bolster the vital long-term commitments of governments and policy makers to implementation of vaccine programmes, in the face of competing needs and finite resources. Shifting the focus of projections of the expected impact of vaccination from a long-term cross-sectional perspective towards a cohort-specific point of view on today's young girls who might develop cervical cancer if not vaccinated is fundamental to reinforcing the commitment and overcoming the hesitancy of key public health stakeholders towards HPV vaccination.^25^

Approximately 50% of all cases are expected to occur before 60 years of age, with the average age at diagnosis higher in Africa, Asia, and Latin America. The relatively young age of occurrence of cervical cancer implies substantial loss of life-years^26^ because of premature death from this disease, as well as considerable economic and societal impact, since women, frequently multiparous, are affected during a highly productive and care-giving phase of their lives.^27^

The global increase in the burden of cervical cancer is driven by the number of cases expected to occur in Africa, Latin America, and central and south Asia, and is due to an overall improved life expectancy and declining neonatal and child mortality.^28^ Such a demographic transition implies substantial changes in the age distribution of cervical cancer cases over time. By contrast, a reduction in the number of cancers is expected to occur in Europe, Oceania, Russia, China, and other high-resource countries in Asia, as a result of decreasing trends in fertility rates.^28^

HPV prevalence reduction in the population is a directly observable measure of the population-level impact of HPV vaccination and results from the combined direct and indirect effect of specific characteristics of a vaccination programme as well as population-specific sexual behaviour. Of note, several different scenarios could lead to similar levels of prevalence reduction.^18^, ^19^ Prevalence reduction can be empirically assessed from local data a few years after the introduction of vaccination.^23^, ^29^ HPV prevalence reduction, attributable to vaccination, is expected to vary across countries. Nevertheless, the impact of HPV vaccination remains significant even in the case of suboptimal scenarios. Therefore, we also report the number of cancer cases prevented in each country with lower levels of prevalence reduction. A direct comparison of these numbers with the expected reduction in the case of highest effectiveness will provide clear indications to public health officials on the need for remedial actions, such as catch-up vaccination campaigns or adaptation of cervical cancer screening algorithms to local contexts.

Our approach to estimate the country-specific expected number of cervical cancers is designed to minimise the assumptions (along with their intrinsic limitations) underlying our projections. First, our projections are based on GLOBOCAN 2018 estimates, which are informed by the best available sources of cancer incidence and mortality data within a given country. Therefore, the validity of the national estimates depends on the degree of representativeness and the quality of source information.^16^, ^17^ Second, country-specific birth-cohort survival is based on life-expectancy assumptions incorporated into the projections issued by the UN Population Division.^28^ Third, to minimise possible distortions due to small local samples, we used supra-national estimates of the relative contributions of each HPV type to cervical cancer burden to estimate the number of cervical cancer cases preventable through vaccination. Fourth, we assume no changes in annual cervical cancer incidence between birth cohorts. This assumption could have led to conservative estimates of the expected number of cancers in countries where sexual behaviour is changing towards less traditional patterns.^30^ For example, data from some urban areas in China show increasing trends in cervical cancer incidence in younger birth cohorts.^31^ Nevertheless, although the method proposed in the present Article can incorporate variations of cervical cancer incidence across birth cohorts, suitable data on national trends in cervical cancer incidence are mostly unavailable. Fifth, we assume no changes in cervical cancer screening practices. This assumption might lead to conservative estimates of the overall number of cancers preventable in countries where cervical cancer screening will be successfully implemented. Indeed, global modelling exercises have clearly illustrated the importance of scaling up vaccination, screening, and treatment for pre-invasive and invasive cervical cancer as rapidly as possible.^12^, ^13^, ^14^ Finally, since very few countries have sufficient high-quality data to reliably characterise the sexual behaviour of their population, our estimates did not take into account such data.

In conclusion, we have assessed the expected burden of cervical cancer in the absence of HPV vaccination and the impact of vaccination from the perspective of today's girls and young women at an eligible age for immunisation who could be protected against cervical cancer during their lifespan. As has been noted elsewhere, lifetime risk is a suitable indicator to inform public health targets,^32^ and we believe the estimation of the expected and preventable cervical cancers among recently born girls is of direct relevance to public health decision makers seeking to assess the impact of the HPV vaccination among today's young girls. These estimations could also serve as an advocacy tool for effective national implementation, given the short-term moral imperative of saving lives through national commitment to HPV vaccination programmes.

## Data sharing

Readers can access the data used in this study from the links to public domain resources provided in the Methods. The code used to generate the reported estimates will be made available upon request to the corresponding author.

## Declaration of interests

ELF has received grants to his university and personal fees from Merck, outside of the submitted work. ELF has a patent “Methylation markers in cervical cancer” pending to his university. The other authors declare no competing interests.
