# Supplementary material for: Global estimates of expected and preventable cervical cancers among girls born between 2005 and 2014: a birth cohort analysis
Source: Lancet Public Health. 2021 Apr 15;6(7):e510–21. doi: 10.1016/S2468-2667(21)00046-3 (PMC8225515; doi:10.1016/S2468-2667(21)00046-3)

THE LANCET
Public Health

Supplementary appendix

This appendix formed part of the original submission and has been peer reviewed. We post it as supplied by the authors.

Supplement to: Bonjour M, Charvat H, Franco EL, et al. Global estimates of expected and preventable cervical cancers among girls born between 2005 and 2014: a birth cohort analysis. *Lancet Public Health* 2021; published online April 14. http://dx.doi.org/10.1016/S2468-2667(21)00046-3.

Appendix

Contents

Technical Appendix: Global estimates of expected and preventable cervical cancers among girls born between 2005-14: a birth cohort analysis. 2

Table S1. Country-specific estimated number of lifetime cervical cancer cases expected in the absence of HPV vaccination and preventable through vaccination among women born between 2005 and 2014 by postulated levels of HPV prevalence reduction, attributable to vaccination with a vaccine targeting HPV16/18 types and offering 50% cross-protection against HPV31/33/35 types. 5

Table S2. Country-specific estimated number of lifetime cervical cancer cases expected in the absence of HPV vaccination and preventable through vaccination among women born between 2005 and 2014 by postulated levels of HPV prevalence reduction, attributable to vaccination with a vaccine targeting HPV16/18 types without cross-protection. 14

Table S3. Country-specific estimated number of lifetime cervical cancer cases expected in the absence of HPV vaccination and preventable through vaccination among women born between 2005 and 2014 by postulated levels of HPV prevalence reduction, attributable to vaccination with a vaccine targeting HPV16/18 and, 31, 33, 45, 52, 58 types. 23

Supplementary Figure 1. Country-specific ratio of the future number of cervical cancers cases expected in the cohort born in year 2005 in the absence of vaccination to the number of cases estimated by GLOBOCAN for year 2018 by human development index. 32

Technical Appendix: Global estimates of expected and preventable cervical cancers among girls born between 2005-14: a birth cohort analysis.

Statistical methods

Derivation of the formula for estimating the contribution to the cumulative incidence of cervical cancer for a particular age group taking account of the competing risk of dying from any cause before being diagnosed with cervical cancer.

For a given cohort of women, the expected cumulative number of cervical cancers between age 15 and 79 years in the absence of vaccination was calculated by using 5-year age group-specific cervical cancer incidence rate projections from the Global Cancer Observatory (GLOBOCAN) (http://gco.iar.fr) project,[1,2] and 5-year age group-specific mortality rates were obtained from United Nations, Department of Economic and Social Affairs, Population Division.[3]

Let A_0 be the age group 15-19 years, …, A_{12} be the age group 75-79 years. Let us denote by λ_C and λ_M respectively cervical cancer incidence rate and mortality rate, both assumed to be piecewise constant functions on each five-year age group. That is, for $i = 0, \ldots, 12$, $\lambda_{C,i}$ and $\lambda_{M,i}$ denote respectively the constant A_i-specific cervical cancer incidence and mortality rates. Let us also designate by A_i^L and A_i^U respectively the lower and upper bound of age interval A_i, with for $i > 0$, $A_i^L = A_{i-1}^U$. Then, the survival free from event (i.e., cervical cancer diagnosis or death) by age A_I^U for a cohort of women aged 15 at the start of follow-up can be calculated according to the formula:

$$S(A_I^U) = S(A_{I+1}^L) = \exp\left(-\int_{A_0^L}^{A_I^U} \big(\lambda_C(u) + \lambda_M(u)\big)du\right)$$

$$= \exp\left(-5\sum_{i=0}^{I}\big(\lambda_{C,i} + \lambda_{M,i}\big)\right)$$

Now, the cumulative incidence of cervical cancer by age A_I^U can be calculated as:

$$CumI(A_I^U) = \int_{A_0^L}^{A_I^U} \lambda_C(u) S(u) du$$

$$= \sum_{i=0}^{I} \lambda_{C,i} \int_{A_i^L}^{A_i^U} S(u) du$$

$$= \sum_{i=0}^{I} CumI_i$$

We then have:

$$CumI_i = \lambda_{C,i} \int_{A_i^L}^{A_i^U} S(u) du$$

$$= \lambda_{C,i} \int_{A_i^L}^{A_i^U} \exp\left(-\int_{A_0^L}^{u} \big(\lambda_C(v) + \lambda_M(v)\big) dv\right) du$$

$$= \lambda_{C,i} \int_{A_i^L}^{A_i^U} \exp\left(-\int_{A_0^L}^{A_i^L} \big(\lambda_C(v) + \lambda_M(v)\big) dv\right) \exp\left(-\int_{A_i^L}^{u} \big(\lambda_C(v) + \lambda_M(v)\big) dv\right) du$$

$$= \lambda_{C,i} \exp\left(-5 \sum_{k=0}^{i-1} \big(\lambda_{C,k} + \lambda_{M,k}\big)\right) \int_{A_i^L}^{A_i^U} \exp\left(-\int_{A_i^L}^{u} \big(\lambda_C(v) + \lambda_M(v)\big) dv\right) du$$

$$= -\frac{\lambda_{C,i}}{\lambda_{C,i} + \lambda_{M,i}} \exp\left(-5 \sum_{k=0}^{i-1} \big(\lambda_{C,k} + \lambda_{M,k}\big)\right) \Big(\exp\Big(-5\big(\lambda_{C,i} + \lambda_{M,i}\big)\Big) - 1\Big)$$

$$= -\frac{\lambda_{C,i}}{\lambda_{C,i} + \lambda_{M,i}} \Big(S\big(A_i^U\big) - S\big(A_{i-1}^U\big)\Big)$$

$$= \frac{\lambda_{C,i}}{\lambda_{C,i} + \lambda_{M,i}} \Big(S\big(A_i^L\big) - S\big(A_i^U\big)\Big)$$

Based on the assumption that the age group-specific incidence rates were stable across birth cohorts, the cumulative incidence between ages 15 and 79 was then obtained by summing the age group-specific contributions:$\mathrm{CumI}_{15-79} = \sum_{i=0}^{12} \mathrm{CumI}_\mathrm{i}$. Finally, the cumulative number of cervical cancers was calculated by

multiplying this cumulative incidence by the size of the cohort at age 15 years, also estimated from the GLOBOCAN data. Uncertainty intervals were estimated with Monte-Carlo simulation combining uncertainty about cervical cancer incidence as reported in GLOBOCAN 2018 and HPV distribution in cervical cancers as reported by Guan et al.[4]

References

1. Bray F, Ferlay J, Soerjomataram I, Siegel RL, Torre LA, Jemal A. Global cancer statistics 2018: GLOBOCAN estimates of incidence and mortality worldwide for 36 cancers in 185 countries. *CA Cancer J Clin* 2018; **68**(6): 394-424.
2. Ferlay J, Colombet M, Soerjomataram I, et al. Estimating the global cancer incidence and mortality in 2018: GLOBOCAN sources and methods. *Int J Cancer* 2019; **144**(8): 1941-53.
3. United Nations Department of Economic and Social Affairs Population Division. World Population Prospects 2019. https://population.un.org/wpp/ (accessed 29 Sept 2020).
4. Guan P, Howell-Jones R, Li N, et al. Human papillomavirus types in 115,789 HPV-positive women: A meta-analysis from cervical infection to cancer. *Int J Cancer* 2012; **131**(10): 2349-59.

Table S1. Country-specific estimated number of lifetime cervical cancer cases expected in the absence of HPV vaccination and preventable through vaccination among women born between 2005 and 2014 by postulated levels of HPV prevalence reduction, attributable to vaccination with a vaccine targeting HPV16/18 types and offering 50% cross-protection against HPV31/33/35 types.

	Estimated number (95% uncertainty intervals) of cancers					
Country (Women at risk)	Expected in the absence of vaccination	Avoided with HPV prevalence reduction, attributable to vaccination				
		60%	70%	80%	90%	100%
Afghanistan (4 993 596)	33 107 (23 680 - 46 648)	15 624 (11 154 - 22 014)	18 228 (13 013 - 25 683)	20 832 (14 872 - 29 352)	23 436 (16 731 - 33 021)	26 040 (18 590 - 36 690)
Albania (158 178)	1138 (754 - 1722)	545 (361 - 825)	636 (422 - 962)	727 (482 - 1100)	817 (542 - 1237)	908 (602 - 1375)
Algeria (3 940 923)	40 170 (35 139 - 45 656)	17 763 (15 545 - 20 243)	20 724 (18 135 - 23 617)	23 684 (20 726 - 26 991)	26 645 (23 317 - 30 365)	29 606 (25 908 - 33 739)
Angola (4 584 705)	171 867 (130 109 - 226 412)	76 001 (57 526 - 100 227)	88 668 (67 114 - 116 932)	101 335 (76 701 - 133 636)	114 002 (86 289 - 150 341)	126 669 (95 877 - 167 045)
Argentina (3 600 658)	61 460 (55 784 - 67 709)	25 875 (23 473 - 28 555)	30 187 (27 385 - 33 314)	34 500 (31 297 - 38 074)	38 812 (35 209 - 42 833)	43 125 (39 121 - 47 592)
Armenia (184 974)	1875 (1179 - 2983)	885 (556 - 1411)	1033 (649 - 1646)	1180 (742 - 1881)	1328 (835 - 2116)	1475 (927 - 2351)
Australia (1 533 702)	8589 (7729 - 9565)	4267 (3825 - 4769)	4978 (4462 - 5564)	5690 (5100 - 6359)	6401 (5737 - 7154)	7112 (6375 - 7949)
Austria (396 884)	2363 (1895 - 2939)	1131 (906 - 1408)	1320 (1057 - 1643)	1509 (1208 - 1877)	1697 (1359 - 2112)	1886 (1510 - 2347)
Azerbaijan (694 232)	5237 (4574 - 5983)	2471 (2152 - 2826)	2883 (2511 - 3297)	3295 (2869 - 3768)	3707 (3228 - 4239)	4119 (3587 - 4710)
Bahamas (26 573)	306 (163 - 547)	129 (69 - 231)	150 (80 - 269)	172 (91 - 308)	193 (103 - 346)	215 (114 - 385)
Bahrain (96 095)	405 (191 - 760)	191 (91 - 358)	223 (106 - 418)	255 (121 - 477)	287 (136 - 537)	318 (151 - 597)
Bangladesh (15 204 318)	182 334 (130 451 - 255 672)	78 370 (56 109 - 109 548)	91 432 (65 461 - 127 806)	104 494 (74 812 - 146 064)	117 556 (84 164 - 164 322)	130 617 (93 515 - 182 580)
Barbados (17 935)	310 (175 - 529)	131 (74 - 223)	152 (86 - 260)	174 (98 - 297)	196 (111 - 334)	218 (123 - 371)
Belarus (511 896)	6136 (5388 - 6979)	2938 (2581 - 3345)	3427 (3011 - 3903)	3917 (3441 - 4460)	4406 (3871 - 5018)	4896 (4302 - 5575)
Belgium (642 474)	5043 (4480 - 5684)	2414 (2144 - 2722)	2817 (2502 - 3176)	3219 (2859 - 3630)	3622 (3216 - 4084)	4024 (3574 - 4537)
Belize (38 143)	1139 (598 - 2024)	480 (252 - 852)	559 (294 - 994)	639 (336 - 1136)	719 (378 - 1278)	799 (420 - 1420)
Benin (1 537 365)	49 686 (41 162 - 59 838)	21 972 (18 199 - 26 478)	25 634 (21 232 - 30 891)	29 296 (24 265 - 35 304)	32 958 (27 299 - 39 717)	36 619 (30 332 - 44 130)

Bhutan (70 699)	1064 (866 - 1287)	457 (372 - 553)	534 (434 - 646)	610 (496 - 738)	686 (558 - 830)	762 (620 - 922)
Bolivia (1 137 316)	42 343 (37 683 - 47 631)	17 826 (15 852 - 20 068)	20 798 (18 494 - 23 413)	23 769 (21 136 - 26 757)	26 740 (23 778 - 30 102)	29 711 (26 420 - 33 447)
Bosnia Herzegovina (162 833)	3487 (2971 - 4082)	1669 (1423 - 1953)	1947 (1661 - 2279)	2226 (1898 - 2605)	2504 (2135 - 2930)	2782 (2372 - 3256)
Botswana (235 572)	8551 (7191 - 10 075)	3781 (3181 - 4458)	4412 (3711 - 5201)	5042 (4241 - 5944)	5672 (4771 - 6687)	6302 (5301 - 7430)
Brazil (14 792 741)	185 226 (174 108 - 196 716)	77 981 (73 187 - 82 950)	90 978 (85 385 - 96 775)	103 975 (97 582 - 110 600)	116 971 (109 780 - 124 425)	129 968 (121 978 - 138 250)
Brunei Darussalam (31 490)	615 (382 - 974)	264 (164 - 419)	308 (192 - 489)	352 (219 - 559)	396 (246 - 629)	440 (274 - 698)
Bulgaria (334 066)	6663 (5906 - 7544)	3190 (2827 - 3613)	3722 (3298 - 4216)	4253 (3769 - 4818)	4785 (4240 - 5420)	5317 (4712 - 6022)
Burkina Faso (2 793 581)	122 203 (48 333 - 307 828)	54 040 (21 387 - 136 046)	63 046 (24 952 - 158 720)	72 053 (28 516 - 181 394)	81 059 (32 081 - 204 068)	90 066 (35 645 - 226 743)
Burundi (1 589 898)	95 457 (62 427 - 142 581)	42 212 (27 664 - 63 015)	49 248 (32 274 - 73 518)	56 283 (36 885 - 84 020)	63 318 (41 496 - 94 523)	70 354 (46 106 - 105 025)
Cambodia (1 633 459)	26 186 (14 597 - 46 518)	11 255 (6282 - 19 997)	13 131 (7329 - 23 330)	15 007 (8376 - 26 663)	16 883 (9423 - 29 995)	18 759 (10 470 - 33 328)
Cameroon (3 344 918)	110 413 (87 675 - 137 693)	48 826 (38 802 - 60 825)	56 963 (45 269 - 70 962)	65 101 (51 737 - 81 100)	73 239 (58 204 - 91 237)	81 376 (64 671 - 101 375)
Canada (1 946 458)	9374 (8191 - 10 722)	4429 (3868 - 5077)	5168 (4513 - 5923)	5906 (5158 - 6769)	6644 (5803 - 7615)	7382 (6447 - 8462)
Cape Verde (54 412)	1444 (793 - 2537)	639 (351 - 1120)	745 (409 - 1306)	851 (468 - 1493)	958 (526 - 1679)	1064 (585 - 1866)
Central African Republic (663 673)	14 910 (11 175 - 19 852)	6593 (4937 - 8789)	7692 (5760 - 10 253)	8791 (6583 - 11 718)	9890 (7406 - 13 183)	10 989 (8229 - 14 648)
Chad (2 263 556)	52 520 (39 479 - 69 548)	23 225 (17 476 - 30 749)	27 096 (20 388 - 35 874)	30 967 (23 301 - 40 999)	34 837 (26 213 - 46 124)	38 708 (29 126 - 51 248)
Chile (1 208 357)	12 326 (10 383 - 14 667)	5189 (4370 - 6173)	6054 (5099 - 7201)	6919 (5827 - 8230)	7784 (6555 - 9259)	8649 (7284 - 10 288)
China (77 115 987)	867 587 (840 459 - 895 528)	372 904 (360 468 - 385 800)	435 055 (420 546 - 450 100)	497 205 (480 624 - 514 400)	559 356 (540 702 - 578 700)	621 507 (600 780 - 643 000)
Colombia (3 768 405)	55 980 (49 589 - 63 180)	23 568 (20 863 - 26 590)	27 496 (24 340 - 31 021)	31 424 (27 817 - 35 453)	35 352 (31 294 - 39 884)	39 280 (34 771 - 44 316)
Comoros (104 310)	6059 (3903 - 9218)	2679 (1726 - 4082)	3126 (2013 - 4762)	3572 (2301 - 5443)	4019 (2589 - 6123)	4466 (2876 - 6804)
Congo, DR (12 152 010)	319 343 (241 012 - 419 998)	141 217 (106 565 - 186 113)	164 753 (124 325 - 217 132)	188 289 (142 086 - 248 150)	211 825 (159 847 - 279 169)	235 361 (177 608 - 310 188)
Congo (Brazzaville) (730 066)	16 571 (12 993 - 21 138)	7328 (5738 - 9369)	8549 (6694 - 10 931)	9770 (7651 - 12 493)	10 992 (8607 - 14 054)	12 213 (9563 - 15 616)
Costa Rica (348 535)	4182 (3113 - 5582)	1761 (1310 - 2350)	2054 (1529 - 2741)	2348 (1747 - 3133)	2641 (1965 - 3524)	2934 (2184 - 3916)
Cote d Ivoire (3 344 463)	121 472 (103 648 - 142 403)	53 716 (45 829 - 63 204)	62 669 (53 467 - 73 738)	71 622 (61 105 - 84 272)	80 575 (68 743 - 94 806)	89 527 (76 381 - 105 341)

Croatia (204 038)	1464 (1132 - 1883)	701 (542 - 902)	818 (633 - 1052)	934 (723 - 1203)	1051 (813 - 1353)	1168 (904 - 1503)
Cuba (586 649)	7700 (6662 - 8891)	3242 (2806 - 3747)	3782 (3273 - 4371)	4323 (3741 - 4996)	4863 (4209 - 5620)	5403 (4676 - 6245)
Cyprus (64 836)	357 (235 - 534)	168 (111 - 252)	197 (129 - 294)	225 (148 - 336)	253 (166 - 378)	281 (185 - 420)
Czechia (543 020)	5627 (4918 - 6442)	2694 (2352 - 3083)	3143 (2744 - 3597)	3592 (3136 - 4111)	4041 (3528 - 4625)	4490 (3920 - 5139)
Denmark (313 564)	3345 (2860 - 3908)	1601 (1368 - 1872)	1868 (1596 - 2184)	2135 (1824 - 2496)	2402 (2052 - 2808)	2669 (2280 - 3120)
Djibouti (96 726)	1534 (948 - 2362)	678 (419 - 1044)	791 (489 - 1218)	904 (558 - 1392)	1017 (628 - 1566)	1130 (698 - 1740)
Dominican Republic (1 032 233)	20 088 (16 448 - 24 491)	8457 (6917 - 10 321)	9867 (8070 - 12 041)	11 276 (9222 - 13 761)	12 686 (10 375 - 15 481)	14 095 (11 528 - 17 202)
Ecuador (1 540 586)	26 933 (24 331 - 29 790)	11 339 (10 243 - 12 551)	13 229 (11 950 - 14 643)	15 118 (13 657 - 16 734)	17 008 (15 364 - 18 826)	18 898 (17 072 - 20 918)
Egypt (10 256 172)	30 537 (22 384 - 41 777)	13 504 (9901 - 18 440)	15 754 (11 552 - 21 514)	18 005 (13 202 - 24 587)	20 256 (14 852 - 27 661)	22 506 (16 502 - 30 734)
El Salvador (566 651)	11 760 (9766 - 14 125)	4951 (4112 - 5944)	5776 (4797 - 6935)	6601 (5483 - 7926)	7427 (6168 - 8916)	8252 (6853 - 9907)
Equatorial Guinea (151 541)	4419 (3195 - 6063)	1954 (1412 - 2680)	2280 (1648 - 3127)	2605 (1883 - 3574)	2931 (2119 - 4020)	3257 (2354 - 4467)
Eritrea (692 925)	10 979 (7134 - 16 771)	4855 (3147 - 7420)	5664 (3672 - 8656)	6473 (4196 - 9893)	7282 (4721 - 11 130)	8091 (5245 - 12 366)
Estonia (71 667)	1379 (1103 - 1725)	660 (528 - 826)	770 (616 - 964)	880 (704 - 1101)	990 (792 - 1239)	1100 (880 - 1377)
Ethiopia (13 813 867)	298 895 (241 982 - 371 142)	132 175 (106 883 - 164 319)	154 204 (124 697 - 191 705)	176 233 (142 511 - 219 092)	198 262 (160 325 - 246 478)	220 291 (178 139 - 273 865)
Fiji (84 107)	2101 (1707 - 2568)	1044 (847 - 1281)	1218 (988 - 1495)	1392 (1129 - 1708)	1566 (1270 - 1922)	1740 (1411 - 2135)
Finland (298 748)	1260 (993 - 1607)	603 (475 - 769)	703 (554 - 897)	804 (633 - 1025)	904 (712 - 1153)	1005 (792 - 1281)
France (3 847 396)	25 943 (23 953 - 28 050)	12 420 (11 462 - 13 432)	14 490 (13 373 - 15 671)	16 560 (15 283 - 17 909)	18 630 (17 193 - 20 148)	20 701 (19 104 - 22 387)
France, Guadeloupe (27 340)	330 (213 - 501)	139 (90 - 211)	162 (105 - 247)	185 (120 - 282)	209 (135 - 317)	232 (150 - 352)
France, La Reunion (67 334)	728 (540 - 968)	322 (239 - 428)	376 (279 - 499)	429 (318 - 571)	483 (358 - 642)	536 (398 - 714)
France, Martinique (23 264)	200 (117 - 316)	84 (49 - 133)	98 (57 - 155)	112 (66 - 177)	126 (74 - 200)	141 (82 - 222)
France, New Caledonia (19 996)	348 (206 - 569)	173 (102 - 284)	202 (119 - 331)	231 (136 - 378)	259 (153 - 426)	288 (170 - 473)
French Guyana (30 677)	738 (373 - 1365)	311 (157 - 575)	362 (183 - 670)	414 (209 - 766)	466 (235 - 862)	518 (261 - 958)
French Polynesia (21 264)	262 (133 - 484)	130 (66 - 240)	152 (77 - 281)	174 (88 - 321)	195 (99 - 361)	217 (110 - 401)

Gabon<br>(236 188)	5594 (4351 - 7049)	2474 (1923 - 3122)	2886 (2244 - 3642)	3298 (2565 - 4162)	3711 (2885 - 4682)	4123 (3206 - 5203)
Georgia<br>(239 957)	2543 (1709 - 3770)	1200 (804 - 1774)	1400 (938 - 2070)	1600 (1072 - 2365)	1800 (1206 - 2661)	2000 (1340 - 2957)
Germany<br>(3 476 839)	26 017 (24 445 - 27 687)	12 455 (11 694 - 13 264)	14 531 (13 643 - 15 474)	16 607 (15 591 - 17 685)	18 683 (17 540 - 19 896)	20 759 (19 489 - 22 106)
Ghana<br>(3 574 916)	126 482 (112 467 - 141 862)	55 932 (49 662 - 62 809)	65 254 (57 939 - 73 278)	74 576 (66 216 - 83 746)	83 898 (74 493 - 94 214)	93 220 (82 770 - 104 682)
Greece<br>(531 559)	4165 (3152 - 5478)	1994 (1506 - 2625)	2326 (1757 - 3063)	2659 (2008 - 3500)	2991 (2259 - 3938)	3323 (2510 - 4375)
Guam<br>(13 054)	251 (193 - 321)	125 (96 - 160)	145 (112 - 186)	166 (128 - 213)	187 (144 - 240)	208 (160 - 266)
Guatemala<br>(1 913 663)	42 917 (37 066 - 49 600)	18 068 (15 590 - 20 882)	21 080 (18 189 - 24 362)	24 091 (20 787 - 27 843)	27 102 (23 385 - 31 323)	30 114 (25 984 - 34 803)
Guinea-Bissau<br>(251 969)	10 682 (4160 - 27 164)	4724 (1841 - 12 054)	5511 (2148 - 14 063)	6298 (2455 - 16 072)	7086 (2762 - 18 081)	7873 (3069 - 20 090)
Guinea<br>(1 749 245)	99 417 (79 879 - 123 716)	43 963 (35 300 - 54 709)	51 290 (41 184 - 63 827)	58 618 (47 067 - 72 945)	65 945 (52 950 - 82 064)	73 272 (58 834 - 91 182)
Guyana<br>(72 043)	2638 (2042 - 3366)	1110 (859 - 1417)	1296 (1002 - 1653)	1481 (1145 - 1889)	1666 (1289 - 2125)	1851 (1432 - 2361)
Haiti<br>(1 182 320)	24 616 (7602 - 75 534)	10 363 (3190 - 31 850)	12 091 (3721 - 37 158)	13 818 (4253 - 42 467)	15 545 (4784 - 47 775)	17 272 (5316 - 53 083)
Honduras<br>(959 120)	20 994 (17 217 - 25 458)	8838 (7239 - 10 703)	10 311 (8446 - 12 487)	11 785 (9652 - 14 271)	13 258 (10 859 - 16 055)	14 731 (12 065 - 17 839)
Hungary<br>(458 141)	7927 (6812 - 9268)	3795 (3263 - 4437)	4428 (3806 - 5176)	5060 (4350 - 5916)	5693 (4894 - 6655)	6325 (5438 - 7394)
Iceland<br>(22 234)	130 (74 - 224)	62 (35 - 107)	73 (41 - 125)	83 (47 - 143)	93 (53 - 161)	104 (59 - 179)
India<br>(117 883 612)	1 803 757 (1 738 203 - 1 873 304)	925 488 (878 891 - 973 411)	1 079 736 (1 025 373 - 1 135 646)	1 233 984 (1 171 855 - 1 297 882)	1 388 232 (1 318 337 - 1 460 117)	1 542 480 (1 464 819 - 1 622 352)
Indonesia<br>(23 396 629)	556 851 (515 003 - 602 033)	239 344 (221 099 - 259 180)	279 235 (257 949 - 302 376)	319 125 (294 799 - 345 573)	359 016 (331 649 - 388 769)	398 907 (368 499 - 431 966)
Iran, Islamic Republic of<br>(6 365 497)	17 005 (15 213 - 18 965)	8025 (7162 - 8967)	9363 (8356 - 10 461)	10 700 (9549 - 11 956)	12 038 (10 743 - 13 450)	13 375 (11 937 - 14 945)
Iraq<br>(4 936 719)	10 341 (8428 - 12 585)	4880 (3975 - 5951)	5694 (4638 - 6942)	6507 (5300 - 7934)	7320 (5963 - 8926)	8134 (6626 - 9918)
Ireland<br>(340 925)	3650 (2825 - 4726)	1747 (1353 - 2263)	2039 (1579 - 2641)	2330 (1804 - 3018)	2621 (2030 - 3395)	2912 (2255 - 3772)
Israel<br>(747 276)	3972 (3200 - 4912)	1874 (1508 - 2323)	2187 (1759 - 2710)	2499 (2010 - 3098)	2812 (2261 - 3485)	3124 (2513 - 3872)
Italy<br>(2 644 557)	17 468 (16 331 - 18 694)	8363 (7815 - 8948)	9757 (9117 - 10 440)	11 151 (10 419 - 11 931)	12 544 (11 722 - 13 423)	13 938 (13 024 - 14 914)
Jamaica<br>(213 952)	6547 (4355 - 9782)	2756 (1835 - 4119)	3215 (2141 - 4806)	3675 (2447 - 5492)	4134 (2753 - 6179)	4594 (3059 - 6865)
Japan<br>(5 360 201)	71 887 (68 512 - 75 442)	30 898 (29 418 - 32 467)	36 048 (34 320 - 37 878)	41 198 (39 223 - 43 289)	46 348 (44 126 - 48 700)	51 497 (49 029 - 54 111)

Jordan (1 122 839)	4069 (2843 - 5721)	1920 (1339 - 2705)	2240 (1563 - 3156)	2560 (1786 - 3607)	2880 (2009 - 4058)	3200 (2232 - 4509)
Kazakhstan (1 651 384)	27 500 (25 911 - 29 210)	12 978 (12 191 - 13 824)	15 141 (14 223 - 16 128)	17 304 (16 255 - 18 432)	19 467 (18 287 - 20 736)	21 630 (20 318 - 23 040)
Kenya (6 630 383)	274 125 (241 202 - 310 540)	121 221 (106 438 - 137 598)	141 424 (124 178 - 160 531)	161 628 (141 918 - 183 464)	181 831 (159 658 - 206 397)	202 035 (177 397 - 229 330)
Korea, North (1 697 972)	15 751 (13 542 - 18 342)	6770 (5822 - 7881)	7898 (6792 - 9195)	9026 (7763 - 10 509)	10 155 (8733 - 11 822)	11 283 (9704 - 13 136)
Korea, South (2 206 345)	18 752 (17 696 - 19 837)	8060 (7595 - 8532)	9403 (8861 - 9954)	10 746 (10 127 - 11 376)	12 090 (11 393 - 12 798)	13 433 (12 659 - 14 220)
Kuwait (280 477)	1294 (732 - 2086)	611 (346 - 983)	713 (403 - 1147)	814 (461 - 1310)	916 (519 - 1474)	1018 (576 - 1638)
Kyrgyzstan (619 807)	13 002 (9793 - 17 291)	6136 (4619 - 8175)	7158 (5389 - 9537)	8181 (6159 - 10 900)	9204 (6929 - 12 262)	10 226 (7698 - 13 625)
Laos (737 729)	9693 (5400 - 17 541)	4166 (2325 - 7545)	4861 (2713 - 8803)	5555 (3101 - 10 060)	6250 (3488 - 11 318)	6944 (3876 - 12 575)
Latvia (99 928)	2354 (1803 - 3054)	1127 (864 - 1462)	1315 (1008 - 1706)	1502 (1152 - 1950)	1690 (1296 - 2193)	1878 (1439 - 2437)
Lebanon (449 624)	2503 (1979 - 3136)	1181 (934 - 1479)	1378 (1090 - 1726)	1575 (1245 - 1972)	1772 (1401 - 2219)	1969 (1557 - 2465)
Lesotho (257 428)	13 973 (10 653 - 18 170)	6179 (4723 - 8028)	7209 (5510 - 9366)	8239 (6298 - 10 704)	9269 (7085 - 12 042)	10 299 (7872 - 13 380)
Liberia (636 436)	29 393 (11 400 - 74 472)	12 998 (5063 - 32 981)	15 164 (5907 - 38 478)	17 330 (6751 - 43 975)	19 497 (7594 - 49 472)	21 663 (8438 - 54 969)
Libya (585 328)	8096 (5625 - 11 596)	3580 (2491 - 5126)	4177 (2907 - 5980)	4774 (3322 - 6834)	5370 (3737 - 7688)	5967 (4152 - 8543)
Lithuania (138 389)	2521 (2159 - 2950)	1207 (1032 - 1411)	1408 (1204 - 1647)	1609 (1376 - 1882)	1810 (1548 - 2117)	2012 (1720 - 2352)
Luxembourg (31 122)	193 (83 - 431)	92 (40 - 206)	108 (47 - 241)	123 (53 - 275)	139 (60 - 309)	154 (66 - 344)
Macedonia (111 363)	1223 (872 - 1734)	586 (417 - 831)	683 (487 - 969)	781 (557 - 1108)	879 (626 - 1246)	976 (696 - 1385)
Madagascar (3 417 425)	152 716 (100 781 - 229 850)	67 533 (44 547 - 101 500)	78 788 (51 972 - 118 417)	90 044 (59 396 - 135 333)	101 299 (66 821 - 152 250)	112 555 (74 245 - 169 167)
Malawi (2 697 638)	204 058 (172 599 - 239 887)	90 236 (76 204 - 106 269)	105 276 (88 905 - 123 981)	120 315 (101 606 - 141 692)	135 355 (114 307 - 159 404)	150 394 (127 007 - 177 115)
Malaysia (2 461 366)	27 359 (25 156 - 29 687)	11 759 (10 799 - 12 769)	13 719 (12 598 - 14 897)	15 679 (14 398 - 17 025)	17 639 (16 198 - 19 153)	19 599 (17 998 - 21 281)
Maldives (32 519)	843 (558 - 1233)	362 (239 - 529)	423 (279 - 618)	483 (319 - 706)	543 (359 - 794)	604 (399 - 882)
Mali (2 848 972)	146 618 (127 997 - 167 492)	64 836 (56 580 - 74 335)	75 642 (66 010 - 86 724)	86 448 (75 440 - 99 113)	97 254 (84 870 - 111 502)	108 060 (94 300 - 123 891)
Malta (19 795)	68 (34 - 134)	33 (16 - 64)	38 (19 - 75)	44 (22 - 85)	49 (24 - 96)	55 (27 - 107)
Mauritania (569 053)	20 975 (8133 - 53 704)	9276 (3594 - 23 751)	10 822 (4193 - 27 709)	12 367 (4791 - 31 668)	13 913 (5390 - 35 626)	15 459 (5989 - 39 584)

Mauritius<br>(76 281)	1061 (745 - 1492)	469 (330 - 660)	547 (385 - 770)	626 (440 - 880)	704 (495 - 990)	782 (550 - 1100)
Mexico<br>(11 184 468)	136 536 (129 575 - 143 997)	57 482 (54 460 - 60 706)	67 062 (63 537 - 70 824)	76 643 (72 613 - 80 941)	86 223 (81 690 - 91 059)	95 804 (90 766 - 101 177)
Moldova<br>(208 559)	3924 (3148 - 4935)	1879 (1508 - 2361)	2192 (1759 - 2755)	2505 (2010 - 3148)	2818 (2262 - 3542)	3131 (2513 - 3935)
Mongolia<br>(295 899)	8056 (6918 - 9335)	3802 (3266 - 4416)	4436 (3810 - 5152)	5069 (4355 - 5888)	5703 (4899 - 6624)	6337 (5443 - 7361)
Montenegro<br>(37 016)	447 (243 - 838)	214 (116 - 401)	250 (135 - 468)	285 (155 - 535)	321 (174 - 602)	357 (193 - 668)
Morocco<br>(3 146 173)	68 650 (60 752 - 77 146)	30 358 (26 836 - 34 192)	35 417 (31 309 - 39 891)	40 477 (35 782 - 45 590)	45 536 (40 255 - 51 288)	50 596 (44 727 - 56 987)
Mozambique<br>(4 328 490)	176 570 (154 681 - 200 559)	78 081 (68 423 - 88 855)	91 095 (79 827 - 103 664)	104 108 (91 231 - 118 473)	117 122 (102 635 - 133 283)	130 135 (114 039 - 148 092)
Myanmar<br>(4 740 555)	105 792 (59 088 - 187 276)	45 471 (25 356 - 80 472)	53 050 (29 582 - 93 884)	60 628 (33 808 - 107 296)	68 207 (38 034 - 120 708)	75 785 (42 260 - 134 120)
Namibia<br>(303 045)	8577 (6777 - 10 719)	3793 (2994 - 4744)	4425 (3493 - 5535)	5057 (3992 - 6326)	5689 (4491 - 7116)	6321 (4990 - 7907)
Nepal<br>(2 962 654)	64 087 (41 908 - 98 502)	27 546 (17 988 - 42 397)	32 137 (20 986 - 49 464)	36 727 (23 984 - 56 530)	41 318 (26 982 - 63 596)	45 909 (29 980 - 70 662)
New Zealand<br>(306 487)	1639 (1248 - 2170)	814 (620 - 1081)	950 (724 - 1261)	1086 (827 - 1441)	1222 (931 - 1621)	1357 (1034 - 1801)
Nicaragua<br>(585 628)	13 579 (10 693 - 17 233)	5717 (4498 - 7265)	6670 (5248 - 8476)	7623 (5998 - 9686)	8575 (6747 - 10 897)	9528 (7497 - 12 108)
Niger<br>(3 415 273)	34 620 (21 387 - 56 301)	15 309 (9448 - 24 856)	17 861 (11 023 - 28 999)	20 412 (12 598 - 33 142)	22 964 (14 173 - 37 284)	25 515 (15 747 - 41 427)
Nigeria<br>(26 738 733)	877 642 (762 504 - 1 009 298)	388 102 (336 935 - 446 807)	452 786 (393 091 - 521 274)	517 470 (449 247 - 595 742)	582 153 (505 403 - 670 210)	646 837 (561 559 - 744 678)
Norway<br>(309 232)	3194 (2692 - 3802)	1529 (1288 - 1821)	1784 (1503 - 2125)	2039 (1717 - 2429)	2294 (1932 - 2732)	2549 (2147 - 3036)
Oman<br>(325 634)	2296 (1455 - 3470)	1084 (687 - 1634)	1264 (802 - 1907)	1445 (917 - 2179)	1625 (1031 - 2452)	1806 (1146 - 2724)
Pakistan<br>(21 803 270)	186 422 (151 201 - 229 735)	80 127 (64 931 - 98 804)	93 482 (75 753 - 115 271)	106 836 (86 575 - 131 739)	120 191 (97 397 - 148 206)	133 545 (108 219 - 164 673)
Palestine<br>(626 350)	1432 (638 - 3037)	676 (301 - 1435)	788 (351 - 1674)	901 (401 - 1913)	1014 (451 - 2152)	1126 (502 - 2391)
Panama<br>(365 327)	7276 (5616 - 9446)	3063 (2366 - 3977)	3574 (2761 - 4640)	4085 (3155 - 5302)	4595 (3549 - 5965)	5106 (3944 - 6628)
Papua New Guinea<br>(950 656)	27 076 (23 440 - 31 229)	13 452 (11 609 - 15 541)	15 694 (13 544 - 18 131)	17 935 (15 478 - 20 721)	20 177 (17 413 - 23 311)	22 419 (19 348 - 25 901)
Paraguay<br>(654 475)	20 921 (17 092 - 25 650)	8808 (7201 - 10 788)	10 276 (8401 - 12 586)	11 744 (9601 - 14 385)	13 212 (10 801 - 16 183)	14 680 (12 001 - 17 981)
Peru<br>(2 862 578)	77 437 (70 922 - 84 325)	32 601 (29 837 - 35 529)	38 035 (34 810 - 41 450)	43 468 (39 782 - 47 372)	48 902 (44 755 - 53 293)	54 335 (49 728 - 59 215)
Philippines<br>(10 749 238)	178 924 (168 681 - 189 932)	76 905 (72 469 - 81 663)	89 722 (84 547 - 95 274)	102 539 (96 626 - 108 884)	115 357 (108 704 - 122 495)	128 174 (120 782 - 136 105)

Poland (1 887 333)	18 113 (16 811 - 19 527)	8672 (8041 - 9359)	10 117 (9382 - 10 919)	11 562 (10 722 - 12 478)	13 007 (12 062 - 14 038)	14 453 (13 402 - 15 598)
Portugal (464 522)	3294 (2985 - 3623)	1577 (1429 - 1735)	1840 (1667 - 2024)	2102 (1905 - 2313)	2365 (2143 - 2603)	2628 (2381 - 2892)
Puerto Rico (212 701)	1942 (1683 - 2229)	817 (708 - 938)	954 (825 - 1094)	1090 (943 - 1250)	1226 (1061 - 1407)	1362 (1179 - 1563)
Qatar (120 073)	595 (270 - 1053)	281 (127 - 499)	328 (148 - 582)	375 (170 - 666)	421 (191 - 749)	468 (212 - 832)
Romania (988 253)	21 036 (20 070 - 22 094)	10 071 (9598 - 10 582)	11 749 (11 198 - 12 345)	13 428 (12 798 - 14 109)	15 106 (14 397 - 15 873)	16 785 (15 997 - 17 636)
Russian Federation (8 138 006)	135 212 (131 924 - 138 545)	64 733 (63 093 - 66 381)	75 521 (73 608 - 77 444)	86 310 (84 124 - 88 507)	97 099 (94 639 - 99 571)	107 888 (105 155 - 110 634)
Rwanda (1 631 965)	59 549 (44 272 - 79 973)	26 333 (19 556 - 35 373)	30 722 (22 815 - 41 268)	35 111 (26 074 - 47 163)	39 500 (29 333 - 53 059)	43 889 (32 593 - 58 954)
Saint Lucia (10 967)	141 (63 - 294)	59 (27 - 124)	69 (31 - 145)	79 (36 - 165)	89 (40 - 186)	99 (44 - 206)
Samoa (23 463)	334 (158 - 678)	166 (78 - 337)	194 (92 - 393)	221 (105 - 449)	249 (118 - 505)	277 (131 - 561)
Sao Tome and Principe (28 676)	794 (200 - 2130)	351 (88 - 942)	409 (103 - 1098)	468 (117 - 1255)	526 (132 - 1412)	585 (147 - 1569)
Saudi Arabia (2 664 744)	6421 (3738 - 11 099)	3030 (1762 - 5235)	3535 (2055 - 6107)	4040 (2349 - 6980)	4545 (2643 - 7852)	5051 (2936 - 8725)
Senegal (2 207 619)	96 409 (37 289 - 241 164)	42 633 (16 470 - 106 441)	49 739 (19 215 - 124 181)	56 844 (21 960 - 141 921)	63 950 (24 705 - 159 661)	71 055 (27 450 - 177 402)
Serbia (468 077)	10 148 (9222 - 11 175)	4859 (4415 - 5350)	5668 (5151 - 6241)	6478 (5887 - 7133)	7288 (6623 - 8025)	8098 (7359 - 8916)
Sierra Leone (1 049 663)	18 315 (16 110 - 20 730)	8099 (7114 - 9174)	9449 (8300 - 10 703)	10 798 (9486 - 12 232)	12 148 (10 671 - 13 761)	13 498 (11 857 - 15 290)
Singapore (283 694)	3218 (2108 - 4903)	1383 (907 - 2105)	1614 (1058 - 2456)	1844 (1209 - 2807)	2075 (1360 - 3157)	2305 (1511 - 3508)
Slovakia (274 945)	4745 (4338 - 5187)	2272 (2076 - 2484)	2650 (2422 - 2898)	3029 (2768 - 3312)	3407 (3114 - 3726)	3786 (3460 - 4140)
Slovenia (102 069)	705 (467 - 1059)	338 (223 - 506)	394 (260 - 590)	450 (298 - 675)	506 (335 - 759)	563 (372 - 843)
Solomon Islands (76 588)	1745 (1297 - 2304)	867 (644 - 1143)	1012 (751 - 1334)	1156 (858 - 1525)	1301 (965 - 1715)	1445 (1073 - 1906)
Somalia (2 203 776)	59 368 (38 611 - 91 443)	26 253 (17 028 - 40 455)	30 629 (19 866 - 47 197)	35 004 (22 704 - 53 939)	39 380 (25 542 - 60 682)	43 755 (28 380 - 67 424)
South Africa (5 408 513)	233 362 (219 697 - 247 286)	103 195 (96 939 - 109 618)	120 394 (113 095 - 127 888)	137 593 (129 252 - 146 158)	154 792 (145 408 - 164 427)	171 991 (161 565 - 182 697)
South Sudan (1 695 917)	49 728 (32 550 - 75 918)	21 990 (14 429 - 33 619)	25 655 (16 834 - 39 222)	29 320 (19 239 - 44 825)	32 985 (21 643 - 50 429)	36 650 (24 048 - 56 032)
Spain (2 272 466)	12 023 (10 837 - 13 330)	5756 (5182 - 6388)	6716 (6046 - 7453)	7675 (6910 - 8518)	8634 (7774 - 9583)	9594 (8637 - 10 647)
Sri Lanka (1 676 421)	11 271 (10 393 - 12 207)	4845 (4462 - 5251)	5652 (5206 - 6127)	6459 (5949 - 7002)	7267 (6693 - 7877)	8074 (7437 - 8752)

Sudan (5 360 470)	54 945 (38 132 - 80 092)	24 297 (16 890 - 35 360)	28 347 (19 704 - 41 253)	32 397 (22 519 - 47 147)	36 446 (25 334 - 53 040)	40 496 (28 149 - 58 934)
Suriname (47 779)	1422 (940 - 2097)	599 (395 - 883)	698 (461 - 1030)	798 (527 - 1177)	898 (593 - 1324)	998 (658 - 1471)
Swaziland (167 683)	9849 (7879 - 12 191)	4355 (3479 - 5392)	5081 (4059 - 6291)	5807 (4639 - 7190)	6533 (5219 - 8088)	7259 (5799 - 8987)
Sweden (568 431)	4699 (4170 - 5302)	2249 (1996 - 2537)	2624 (2329 - 2959)	2999 (2662 - 3382)	3374 (2994 - 3805)	3749 (3327 - 4228)
Switzerland (407 313)	1671 (1439 - 1927)	800 (690 - 923)	933 (805 - 1077)	1066 (919 - 1230)	1200 (1034 - 1384)	1333 (1149 - 1538)
Syrian Arab Republic (2 217 214)	8701 (4154 - 18 464)	4106 (1962 - 8753)	4791 (2289 - 10 211)	5475 (2616 - 11 670)	6159 (2943 - 13 129)	6844 (3270 - 14 588)
Tajikistan (1 009 789)	5965 (4190 - 8453)	2815 (1979 - 3990)	3284 (2309 - 4655)	3753 (2638 - 5320)	4222 (2968 - 5985)	4691 (3298 - 6650)
Tanzania (8 367 875)	573 126 (475 687 - 688 429)	253 442 (210 044 - 305 174)	295 683 (245 051 - 356 037)	337 923 (280 058 - 406 899)	380 163 (315 066 - 457 761)	422 404 (350 073 - 508 624)
Thailand (3 911 333)	57 109 (54 689 - 59 742)	24 547 (23 468 - 25 717)	28 638 (27 379 - 30 003)	32 729 (31 291 - 34 289)	36 820 (35 202 - 38 575)	40 911 (39 113 - 42 861)
The Gambia (307 388)	8630 (4968 - 14 886)	3816 (2201 - 6567)	4452 (2568 - 7661)	5088 (2934 - 8755)	5724 (3301 - 9850)	6361 (3668 - 10 944)
The Netherlands (904 567)	4914 (4277 - 5646)	2352 (2046 - 2704)	2745 (2387 - 3155)	3137 (2728 - 3605)	3529 (3069 - 4056)	3921 (3410 - 4507)
Timor-Leste (183 199)	2411 (1345 - 4319)	1036 (579 - 1855)	1209 (676 - 2164)	1382 (772 - 2473)	1554 (869 - 2782)	1727 (965 - 3091)
Togo (1 066 033)	26 566 (23 809 - 29 538)	11 748 (10 518 - 13 094)	13 706 (12 271 - 15 277)	15 664 (14 024 - 17 459)	17 622 (15 777 - 19 641)	19 580 (17 530 - 21 824)
Trinidad and Tobago (93 918)	1252 (764 - 2047)	527 (321 - 861)	615 (375 - 1005)	703 (428 - 1149)	791 (482 - 1292)	878 (535 - 1436)
Tunisia (885 346)	3353 (2684 - 4206)	1483 (1187 - 1861)	1730 (1385 - 2172)	1977 (1583 - 2482)	2224 (1781 - 2792)	2471 (1978 - 3102)
Turkey (6 593 543)	31 841 (28 759 - 35 356)	15 027 (13 551 - 16 714)	17 531 (15 810 - 19 499)	20 035 (18 069 - 22 285)	22 540 (20 327 - 25 071)	25 044 (22 586 - 27 856)
Turkmenistan (565 752)	7983 (5969 - 10 566)	3767 (2811 - 4990)	4395 (3280 - 5821)	5023 (3748 - 6653)	5651 (4217 - 7485)	6279 (4686 - 8316)
Uganda (6 620 397)	378 424 (331 213 - 431 161)	167 343 (146 239 - 190 825)	195 234 (170 612 - 222 629)	223 124 (194 985 - 254 433)	251 015 (219 358 - 286 237)	278 905 (243 732 - 318 041)
Ukraine (2 264 891)	33 354 (31 732 - 35 075)	15 968 (15 170 - 16 791)	18 629 (17 698 - 19 590)	21 291 (20 226 - 22 388)	23 952 (22 755 - 25 187)	26 614 (25 283 - 27 986)
United Arab Emirates (435 202)	4023 (2540 - 5912)	1898 (1198 - 2788)	2215 (1397 - 3253)	2531 (1597 - 3718)	2848 (1796 - 4182)	3164 (1996 - 4647)
United Kingdom (3 853 641)	29 198 (27 556 - 30 943)	13 978 (13 195 - 14 825)	16 308 (15 395 - 17 296)	18 638 (17 594 - 19 767)	20 968 (19 793 - 22 238)	23 297 (21 992 - 24 709)
United States of America (20 177 675)	131 587 (128 403 - 134 848)	62 176 (60 353 - 64 048)	72 539 (70 412 - 74 723)	82 901 (80 471 - 85 397)	93 264 (90 529 - 96 072)	103 626 (100 588 - 106 747)
Uruguay (237 403)	2880 (2285 - 3576)	1212 (964 - 1506)	1415 (1125 - 1757)	1617 (1286 - 2009)	1819 (1446 - 2260)	2021 (1607 - 2511)

Uzbekistan (2 900 550)	30 906 (28 463 - 33 496)	14 585 (13 422 - 15 853)	17 016 (15 659 - 18 496)	19 447 (17 895 - 21 138)	21 878 (20 132 - 23 780)	24 308 (22 369 - 26 422)
Vanuatu (31 810)	509 (278 - 900)	253 (138 - 447)	295 (161 - 522)	337 (184 - 596)	379 (207 - 671)	421 (230 - 746)
Venezuela (2 879 646)	71 413 (65 650 - 77 710)	30 065 (27 611 - 32 753)	35 076 (32 213 - 38 211)	40 087 (36 815 - 43 670)	45 098 (41 417 - 49 129)	50 109 (46 019 - 54 588)
Vietnam (6 992 903)	54 988 (50 414 - 60 134)	23 635 (21 633 - 25 853)	27 574 (25 239 - 30 162)	31 513 (28 844 - 34 471)	35 452 (32 450 - 38 780)	39 391 (36 055 - 43 089)
Yemen (3 640 898)	7781 (5102 - 11 613)	3672 (2407 - 5485)	4284 (2808 - 6399)	4896 (3209 - 7313)	5508 (3610 - 8227)	6120 (4011 - 9141)
Zambia (2 485 403)	181 270 (157 293 - 208 532)	80 160 (69 439 - 92 277)	93 520 (81 013 - 107 657)	106 880 (92 586 - 123 036)	120 240 (104 159 - 138 416)	133 599 (115 732 - 153 795)
Zimbabwe (2 238 806)	157 452 (143 030 - 173 330)	69 627 (63 077 - 76 781)	81 232 (73 589 - 89 578)	92 836 (84 102 - 102 375)	104 441 (94 615 - 115 172)	116 045 (105 128 - 127 968)

Table S2. Country-specific estimated number of lifetime cervical cancer cases expected in the absence of HPV vaccination and preventable through vaccination among women born between 2005 and 2014 by postulated levels of HPV prevalence reduction, attributable to vaccination with a vaccine targeting HPV16/18 types without cross-protection.

	Estimated number (95% uncertainty intervals) of cancers					
**Country (Women at risk)**	**Expected in the absence of vaccination**	**Avoided with HPV prevalence reduction, attributable to vaccination**				
		**60%**	**70%**	**80%**	**90%**	**100%**
Afghanistan (4 993 596)	33 107 (23 680 - 46 648)	14 408 (10 283 - 20 300)	16 809 (11 997 - 23 683)	19 211 (13 711 - 27 066)	21 612 (15 425 - 30 449)	24 013 (17 139 - 33 833)
Albania (158 178)	1138 (754 - 1722)	503 (333 - 762)	586 (389 - 889)	670 (445 - 1016)	754 (500 - 1142)	838 (556 - 1269)
Algeria (3 940 923)	40 170 (35 139 - 45 656)	15 894 (13 889 - 18 128)	18 543 (16 203 - 21 149)	21 192 (18 518 - 24 170)	23 841 (20 833 - 27 191)	26 491 (23 148 - 30 213)
Angola (4 584 705)	171 867 (130 109 - 226 412)	68 004 (51 472 - 89 621)	79 339 (60 051 - 104 557)	90 673 (68 630 - 119 494)	102 007 (77 208 - 134 431)	113 341 (85 787 - 149 368)
Argentina (3 600 658)	61 460 (55 784 - 67 709)	23 352 (21 174 - 25 775)	27 244 (24 703 - 30 071)	31 136 (28 232 - 34 367)	35 027 (31 761 - 38 662)	38 919 (35 290 - 42 958)
Armenia (184 974)	1875 (1179 - 2983)	816 (512 - 1303)	952 (598 - 1520)	1088 (683 - 1737)	1224 (768 - 1954)	1360 (854 - 2172)
Australia (1 533 702)	8589 (7729 - 9565)	4042 (3612 - 4527)	4716 (4214 - 5282)	5390 (4816 - 6036)	6064 (5418 - 6791)	6737 (6020 - 7546)
Austria (396 884)	2363 (1895 - 2939)	1044 (835 - 1298)	1217 (974 - 1515)	1391 (1113 - 1731)	1565 (1252 - 1948)	1739 (1391 - 2164)
Azerbaijan (694 232)	5237 (4574 - 5983)	2279 (1984 - 2607)	2659 (2315 - 3042)	3039 (2645 - 3476)	3418 (2976 - 3911)	3798 (3307 - 4345)
Bahamas (26 573)	306 (163 - 547)	116 (62 - 208)	136 (72 - 243)	155 (83 - 277)	174 (93 - 312)	194 (103 - 347)
Bahrain (96 095)	405 (191 - 760)	176 (84 - 331)	206 (98 - 386)	235 (111 - 441)	264 (125 - 496)	294 (139 - 551)
Bangladesh (15 204 318)	182 334 (130 451 - 255 672)	72 974 (52 267 - 102 032)	85 136 (60 978 - 119 038)	97 299 (69 689 - 136 043)	109 461 (78 400 - 153 048)	121 623 (87 111 - 170 054)
Barbados (17 935)	310 (175 - 529)	118 (67 - 201)	138 (78 - 235)	157 (89 - 268)	177 (100 - 302)	197 (111 - 335)
Belarus (511 896)	6136 (5388 - 6979)	2709 (2380 - 3086)	3161 (2776 - 3600)	3612 (3173 - 4115)	4064 (3570 - 4629)	4516 (3966 - 5143)
Belgium (642 474)	5043 (4480 - 5684)	2227 (1977 - 2510)	2598 (2306 - 2928)	2969 (2636 - 3346)	3340 (2965 - 3765)	3711 (3295 - 4183)
Belize (38 143)	1139 (598 - 2024)	433 (227 - 769)	505 (265 - 897)	577 (303 - 1026)	649 (341 - 1154)	721 (379 - 1282)
Benin (1 537 365)	49 686 (41 162 - 59 838)	19 660 (16 237 - 23 741)	22 936 (18 943 - 27 698)	26 213 (21 649 - 31 655)	29 490 (24 355 - 35 612)	32 766 (27 061 - 39 568)
Bhutan (70 699)	1064 (866 - 1287)	426 (346 - 515)	497 (404 - 601)	568 (462 - 687)	639 (520 - 773)	710 (577 - 859)

Bolivia (1 137 316)	42 343 (37 683 - 47 631)	16 088 (14 304 - 18 101)	18 769 (16 688 - 21 118)	21 451 (19 072 - 24 135)	24 132 (21 456 - 27 152)	26 814 (23 841 - 30 168)
Bosnia Herzegovina (162 833)	3487 (2971 - 4082)	1539 (1312 - 1802)	1796 (1531 - 2102)	2053 (1750 - 2402)	2309 (1968 - 2703)	2566 (2187 - 3003)
Botswana (235 572)	8551 (7191 - 10 075)	3383 (2848 - 3994)	3947 (3323 - 4660)	4511 (3797 - 5326)	5075 (4272 - 5992)	5639 (4747 - 6657)
Brazil (14 792 741)	185 226 (174 108 - 196 716)	70 377 (66 029 - 74 941)	82 106 (77 034 - 87 432)	93 835 (88 039 - 99 922)	105 565 (99 044 - 112 412)	117 294 (110 049 - 124 902)
Brunei Darussalam (31 490)	615 (382 - 974)	246 (153 - 390)	287 (178 - 455)	328 (204 - 520)	369 (229 - 585)	410 (255 - 650)
Bulgaria (334 066)	6663 (5906 - 7544)	2942 (2607 - 3333)	3433 (3042 - 3888)	3923 (3476 - 4444)	4413 (3911 - 4999)	4904 (4345 - 5555)
Burkina Faso (2 793 581)	122 203 (48 333 - 307 828)	48 353 (19 130 - 121 795)	56 412 (22 319 - 142 094)	64 471 (25 507 - 162 393)	72 530 (28 695 - 182 692)	80 589 (31 884 - 202 991)
Burundi (1 589 898)	95 457 (62 427 - 142 581)	37 771 (24 776 - 56 364)	44 066 (28 906 - 65 758)	50 361 (33 035 - 75 152)	56 656 (37 165 - 84 546)	62 951 (41 294 - 93 940)
Cambodia (1 633 459)	26 186 (14 597 - 46 518)	10 480 (5841 - 18 617)	12 227 (6814 - 21 719)	13 974 (7788 - 24 822)	15 721 (8761 - 27 925)	17 467 (9735 - 31 028)
Cameroon (3 344 918)	110 413 (87 675 - 137 693)	43 688 (34 698 - 54 433)	50 970 (40 482 - 63 505)	58 251 (46 265 - 72 577)	65 532 (52 048 - 81 649)	72 814 (57 831 - 90 721)
Canada (1 946 458)	9374 (8191 - 10 722)	4110 (3586 - 4714)	4796 (4184 - 5500)	5481 (4782 - 6286)	6166 (5380 - 7072)	6851 (5977 - 7857)
Cape Verde (54 412)	1444 (793 - 2537)	571 (313 - 999)	667 (365 - 1166)	762 (417 - 1332)	857 (469 - 1499)	952 (522 - 1665)
Central African Republic (663 673)	14 910 (11 175 - 19 852)	5900 (4413 - 7876)	6883 (5149 - 9189)	7866 (5884 - 10 502)	8849 (6620 - 11 815)	9833 (7355 - 13 127)
Chad (2 263 556)	52 520 (39 479 - 69 548)	20 781 (15 628 - 27 542)	24 245 (18 232 - 32 133)	27 708 (20 837 - 36 723)	31 172 (23 442 - 41 314)	34 635 (26 046 - 45 904)
Chile (1 208 357)	12 326 (10 383 - 14 667)	4683 (3942 - 5570)	5464 (4600 - 6499)	6244 (5257 - 7427)	7025 (5914 - 8356)	7806 (6571 - 9284)
China (77 115 987)	867 587 (840 459 - 895 528)	347 227 (335 406 - 359 352)	405 098 (391 307 - 419 244)	462 969 (447 209 - 479 137)	520 840 (503 110 - 539 029)	578 712 (559 011 - 598 921)
Colombia (3 768 405)	55 980 (49 589 - 63 180)	21 270 (18 828 - 24 019)	24 815 (21 966 - 28 022)	28 360 (25 104 - 32 025)	31 904 (28 242 - 36 028)	35 449 (31 380 - 40 031)
Comoros (104 310)	6059 (3903 - 9218)	2397 (1542 - 3654)	2797 (1799 - 4262)	3197 (2055 - 4871)	3596 (2312 - 5480)	3996 (2569 - 6089)
Congo, DR (12 152 010)	319 343 (241 012 - 419 998)	126 358 (95 226 - 166 293)	147 418 (111 097 - 194 008)	168 477 (126 968 - 221 724)	189 537 (142 839 - 249 439)	210 597 (158 710 - 277 155)
Congo (Brazzaville) (730 066)	16 571 (12 993 - 21 138)	6557 (5137 - 8386)	7650 (5993 - 9784)	8742 (6850 - 11 181)	9835 (7706 - 12 579)	10 928 (8562 - 13 977)
Costa Rica (348 535)	4182 (3113 - 5582)	1589 (1182 - 2123)	1854 (1379 - 2477)	2119 (1576 - 2831)	2383 (1773 - 3184)	2648 (1970 - 3538)
Cote d Ivoire (3 344 463)	121 472 (103 648 - 142 403)	48 064 (40 986 - 56 671)	56 075 (47 817 - 66 116)	64 086 (54 648 - 75 561)	72 096 (61 479 - 85 006)	80 107 (68 310 - 94 451)
Croatia (204 038)	1464 (1132 - 1883)	646 (500 - 833)	754 (583 - 971)	862 (667 - 1110)	970 (750 - 1249)	1077 (833 - 1388)

Cuba (586 649)	7700 (6662 - 8891)	2926 (2530 - 3384)	3413 (2951 - 3948)	3901 (3373 - 4512)	4389 (3795 - 5076)	4876 (4216 - 5640)
Cyprus (64 836)	357 (235 - 534)	155 (102 - 232)	181 (119 - 271)	207 (136 - 309)	233 (153 - 348)	259 (170 - 387)
Czechia (543 020)	5627 (4918 - 6442)	2485 (2169 - 2844)	2899 (2530 - 3318)	3313 (2891 - 3791)	3727 (3253 - 4265)	4141 (3614 - 4739)
Denmark (313 564)	3345 (2860 - 3908)	1477 (1261 - 1727)	1723 (1471 - 2014)	1969 (1681 - 2302)	2216 (1892 - 2590)	2462 (2102 - 2878)
Djibouti (96 726)	1534 (948 - 2362)	607 (374 - 936)	708 (437 - 1092)	809 (499 - 1249)	910 (562 - 1405)	1011 (624 - 1561)
Dominican Republic (1 032 233)	20 088 (16 448 - 24 491)	7632 (6243 - 9323)	8904 (7283 - 10 877)	10 177 (8323 - 12 431)	11 449 (9364 - 13 985)	12 721 (10 404 - 15 539)
Ecuador (1 540 586)	26 933 (24 331 - 29 790)	10 233 (9241 - 11 329)	11 939 (10 781 - 13 217)	13 644 (12 321 - 15 105)	15 350 (13 862 - 16 993)	17 055 (15 402 - 18 881)
Egypt (10 256 172)	30 537 (22 384 - 41 777)	12 083 (8833 - 16 482)	14 097 (10 305 - 19 228)	16 111 (11 777 - 21 975)	18 124 (13 249 - 24 722)	20 138 (14 721 - 27 469)
El Salvador (566 651)	11 760 (9766 - 14 125)	4468 (3709 - 5370)	5213 (4327 - 6265)	5958 (4946 - 7160)	6702 (5564 - 8055)	7447 (6182 - 8950)
Equatorial Guinea (151 541)	4419 (3195 - 6063)	1748 (1261 - 2396)	2040 (1471 - 2795)	2331 (1681 - 3194)	2623 (1891 - 3594)	2914 (2101 - 3993)
Eritrea (692 925)	10 979 (7134 - 16 771)	4344 (2814 - 6650)	5068 (3283 - 7758)	5792 (3752 - 8866)	6516 (4221 - 9974)	7240 (4690 - 11 083)
Estonia (71 667)	1379 (1103 - 1725)	609 (487 - 762)	710 (568 - 889)	812 (650 - 1016)	913 (731 - 1143)	1015 (812 - 1270)
Ethiopia (13 813 867)	298 895 (241 982 - 371 142)	118 267 (95 598 - 147 266)	137 978 (111 531 - 171 810)	157 690 (127 464 - 196 355)	177 401 (143 397 - 220 899)	197 112 (159 330 - 245 443)
Fiji (84 107)	2101 (1707 - 2568)	989 (800 - 1216)	1154 (934 - 1418)	1319 (1067 - 1621)	1483 (1200 - 1823)	1648 (1334 - 2026)
Finland (298 748)	1260 (993 - 1607)	556 (438 - 709)	649 (511 - 828)	742 (584 - 946)	834 (657 - 1064)	927 (731 - 1182)
France (3 847 396)	25 943 (23 953 - 28 050)	11 455 (10 572 - 12 393)	13 365 (12 334 - 14 459)	15 274 (14 096 - 16 525)	17 183 (15 857 - 18 590)	19 092 (17 619 - 20 656)
France, Guadeloupe (27 340)	330 (213 - 501)	126 (81 - 191)	146 (95 - 223)	167 (108 - 254)	188 (122 - 286)	209 (135 - 318)
France, La Reunion (67 334)	728 (540 - 968)	288 (214 - 383)	336 (249 - 447)	384 (285 - 511)	432 (320 - 575)	480 (356 - 639)
France, Martinique (23 264)	200 (117 - 316)	76 (44 - 120)	89 (52 - 140)	101 (59 - 160)	114 (67 - 180)	127 (74 - 200)
France, New Caledonia (19 996)	348 (206 - 569)	164 (97 - 269)	191 (113 - 313)	218 (129 - 358)	246 (145 - 403)	273 (161 - 448)
French Guyana (30 677)	738 (373 - 1365)	280 (142 - 518)	327 (165 - 605)	374 (189 - 691)	421 (213 - 778)	467 (236 - 864)
French Polynesia (21 264)	262 (133 - 484)	123 (63 - 228)	144 (73 - 266)	164 (84 - 304)	185 (94 - 342)	206 (104 - 381)
Gabon (236 188)	5594 (4351 - 7049)	2214 (1716 - 2795)	2582 (2002 - 3261)	2951 (2288 - 3726)	3320 (2574 - 4192)	3689 (2860 - 4658)

Georgia (239 957)	2543 (1709 - 3770)	1107 (744 - 1638)	1291 (868 - 1910)	1476 (992 - 2183)	1660 (1116 - 2456)	1845 (1240 - 2729)
Germany (3 476 839)	26 017 (24 445 - 27 687)	11 488 (10 790 - 12 244)	13 402 (12 588 - 14 285)	15 317 (14 387 - 16 326)	17 232 (16 185 - 18 366)	19 146 (17 983 - 20 407)
Ghana (3 574 916)	126 482 (112 467 - 141 862)	50 047 (44 352 - 56 243)	58 388 (51 744 - 65 617)	66 729 (59 136 - 74 991)	75 070 (66 528 - 84 365)	83 411 (73 920 - 93 739)
Greece (531 559)	4165 (3152 - 5478)	1839 (1389 - 2421)	2146 (1621 - 2824)	2452 (1852 - 3228)	2759 (2084 - 3631)	3065 (2315 - 4035)
Guam (13 054)	251 (193 - 321)	118 (91 - 152)	138 (106 - 177)	157 (121 - 202)	177 (136 - 228)	197 (151 - 253)
Guatemala (1 913 663)	42 917 (37 066 - 49 600)	16 306 (14 053 - 18 847)	19 024 (16 395 - 21 988)	21 742 (18 737 - 25 129)	24 460 (21 079 - 28 270)	27 177 (23 421 - 31 411)
Guinea-Bissau (251 969)	10 682 (4160 - 27 164)	4227 (1645 - 10 772)	4931 (1920 - 12 568)	5636 (2194 - 14 363)	6340 (2468 - 16 158)	7045 (2742 - 17 954)
Guinea (1 749 245)	99 417 (79 879 - 123 716)	39 337 (31 571 - 48 968)	45 894 (36 832 - 57 130)	52 450 (42 094 - 65 291)	59 006 (47 356 - 73 453)	65 562 (52 618 - 81 614)
Guyana (72 043)	2638 (2042 - 3366)	1002 (775 - 1278)	1169 (904 - 1491)	1336 (1033 - 1704)	1503 (1162 - 1917)	1670 (1291 - 2130)
Haiti (1 182 320)	24 616 (7602 - 75 534)	9353 (2886 - 28 755)	10 912 (3367 - 33 548)	12 470 (3848 - 38 340)	14 029 (4329 - 43 133)	15 588 (4810 - 47 926)
Honduras (959 120)	20 994 (17 217 - 25 458)	7977 (6535 - 9675)	9306 (7624 - 11 287)	10 635 (8714 - 12 900)	11 965 (9803 - 14 512)	13 294 (10 892 - 16 124)
Hungary (458 141)	7927 (6812 - 9268)	3500 (3008 - 4095)	4084 (3509 - 4778)	4667 (4010 - 5460)	5251 (4512 - 6143)	5834 (5013 - 6825)
Iceland (22 234)	130 (74 - 224)	57 (33 - 99)	67 (38 - 116)	76 (43 - 132)	86 (49 - 149)	96 (54 - 165)
India (117 883 612)	1 803 757 (1 738 203 - 1 873 304)	881 680 (831 229 - 932 476)	1 028 627 (969 767 - 1 087 889)	1 175 573 (1 108 305 - 1 243 302)	1 322 520 (1 246 843 - 1 398 714)	1 469 467 (1 385 381 - 1 554 127)
Indonesia (23 396 629)	556 851 (515 003 - 602 033)	222 864 (205 784 - 241 465)	260 008 (240 081 - 281 710)	297 151 (274 378 - 321 954)	334 295 (308 675 - 362 198)	371 439 (342 973 - 402 442)
Iran, Islamic Republic of (6 365 497)	17 005 (15 213 - 18 965)	7401 (6591 - 8275)	8634 (7690 - 9654)	9868 (8788 - 11 034)	11 101 (9887 - 12 413)	12 335 (10 985 - 13 792)
Iraq (4 936 719)	10 341 (8428 - 12 585)	4500 (3671 - 5490)	5250 (4283 - 6404)	6001 (4895 - 7319)	6751 (5507 - 8234)	7501 (6119 - 9149)
Ireland (340 925)	3650 (2825 - 4726)	1612 (1251 - 2087)	1880 (1459 - 2434)	2149 (1667 - 2782)	2417 (1876 - 3130)	2686 (2084 - 3478)
Israel (747 276)	3972 (3200 - 4912)	1729 (1388 - 2143)	2017 (1620 - 2500)	2305 (1851 - 2857)	2593 (2082 - 3214)	2881 (2314 - 3572)
Italy (2 644 557)	17 468 (16 331 - 18 694)	7713 (7209 - 8255)	8999 (8410 - 9630)	10 284 (9612 - 11 006)	11 570 (10 813 - 12 382)	12 855 (12 015 - 13 758)
Jamaica (213 952)	6547 (4355 - 9782)	2487 (1654 - 3716)	2902 (1930 - 4335)	3316 (2206 - 4954)	3731 (2481 - 5573)	4146 (2757 - 6193)
Japan (5 360 201)	71 887 (68 512 - 75 442)	28 771 (27 371 - 30 243)	33 566 (31 933 - 35 284)	38 361 (36 494 - 40 324)	43 156 (41 056 - 45 365)	47 951 (45 618 - 50 405)
Jordan (1 122 839)	4069 (2843 - 5721)	1771 (1235 - 2499)	2066 (1440 - 2915)	2361 (1646 - 3332)	2656 (1852 - 3748)	2951 (2058 - 4165)

Kazakhstan<br>(1 651 384)	27 500 (25 911 - 29 210)	11 968 (11 225 - 12 776)	13 962 (13 096 - 14 906)	15 957 (14 967 - 17 035)	17 952 (16 838 - 19 164)	19 946 (18 709 - 21 294)
Kenya<br>(6 630 383)	274 125 (241 202 - 310 540)	108 466 (95 094 - 123 143)	126 544 (110 943 - 143 667)	144 621 (126 792 - 164 190)	162 699 (142 641 - 184 714)	180 777 (158 489 - 205 238)
Korea, North<br>(1 697 972)	15 751 (13 542 - 18 342)	6304 (5416 - 7338)	7354 (6319 - 8561)	8405 (7221 - 9784)	9456 (8124 - 11 007)	10 506 (9027 - 12 230)
Korea, South<br>(2 206 345)	18 752 (17 696 - 19 837)	7505 (7068 - 7952)	8756 (8246 - 9277)	10 006 (9423 - 10 602)	11 257 (10 601 - 11 927)	12 508 (11 779 - 13 253)
Kuwait<br>(280 477)	1294 (732 - 2086)	563 (318 - 906)	657 (371 - 1057)	751 (424 - 1208)	845 (477 - 1359)	939 (530 - 1510)
Kyrgyzstan<br>(619 807)	13 002 (9793 - 17 291)	5658 (4258 - 7537)	6601 (4967 - 8793)	7544 (5677 - 10 049)	8487 (6386 - 11 305)	9431 (7096 - 12 561)
Laos<br>(737 729)	9693 (5400 - 17 541)	3879 (2163 - 7030)	4526 (2523 - 8202)	5173 (2884 - 9373)	5819 (3244 - 10 545)	6466 (3605 - 11 717)
Latvia<br>(99 928)	2354 (1803 - 3054)	1039 (797 - 1350)	1212 (930 - 1575)	1386 (1063 - 1800)	1559 (1196 - 2025)	1732 (1328 - 2250)
Lebanon<br>(449 624)	2503 (1979 - 3136)	1089 (860 - 1365)	1271 (1004 - 1593)	1452 (1147 - 1820)	1634 (1290 - 2048)	1816 (1434 - 2275)
Lesotho<br>(257 428)	13 973 (10 653 - 18 170)	5529 (4224 - 7174)	6450 (4928 - 8370)	7372 (5632 - 9566)	8293 (6336 - 10 761)	9215 (7039 - 11 957)
Liberia<br>(636 436)	29 393 (11 400 - 74 472)	11 630 (4530 - 29 608)	13 568 (5285 - 34 542)	15 507 (6040 - 39 477)	17 445 (6795 - 44 412)	19 384 (7550 - 49 346)
Libya<br>(585 328)	8096 (5625 - 11 596)	3203 (2229 - 4596)	3737 (2600 - 5362)	4271 (2972 - 6128)	4805 (3343 - 6894)	5339 (3715 - 7660)
Lithuania<br>(138 389)	2521 (2159 - 2950)	1113 (952 - 1301)	1299 (1111 - 1518)	1484 (1269 - 1735)	1670 (1428 - 1952)	1855 (1587 - 2169)
Luxembourg<br>(31 122)	193 (83 - 431)	85 (37 - 190)	99 (43 - 222)	114 (49 - 254)	128 (55 - 286)	142 (61 - 317)
Macedonia<br>(111 363)	1223 (872 - 1734)	540 (385 - 766)	630 (449 - 894)	720 (513 - 1022)	810 (577 - 1149)	900 (642 - 1277)
Madagascar<br>(3 417 425)	152 716 (100 781 - 229 850)	60 427 (39 974 - 90 787)	70 498 (46 637 - 105 918)	80 569 (53 299 - 121 050)	90 640 (59 962 - 136 181)	100 712 (66 624 - 151 312)
Malawi<br>(2 697 638)	204 058 (172 599 - 239 887)	80 742 (68 146 - 95 272)	94 199 (79 504 - 111 151)	107 656 (90 862 - 127 029)	121 113 (102 219 - 142 908)	134 569 (113 577 - 158 787)
Malaysia<br>(2 461 366)	27 359 (25 156 - 29 687)	10 950 (10 053 - 11 892)	12 775 (11 728 - 13 874)	14 599 (13 404 - 15 857)	16 424 (15 079 - 17 839)	18 249 (16 755 - 19 821)
Maldives<br>(32 519)	843 (558 - 1233)	337 (223 - 493)	394 (260 - 575)	450 (297 - 657)	506 (335 - 739)	562 (372 - 822)
Mali<br>(2 848 972)	146 618 (127 997 - 167 492)	58 014 (50 587 - 66 498)	67 683 (59 019 - 77 581)	77 352 (67 450 - 88 664)	87 021 (75 881 - 99 747)	96 690 (84 312 - 110 830)
Malta<br>(19 795)	68 (34 - 134)	30 (15 - 59)	35 (17 - 69)	40 (20 - 79)	45 (22 - 89)	50 (25 - 99)
Mauritania<br>(569 053)	20 975 (8133 - 53 704)	8300 (3211 - 21 299)	9683 (3746 - 24 848)	11 066 (4281 - 28 398)	12 449 (4816 - 31 948)	13 833 (5352 - 35 498)
Mauritius<br>(76 281)	1061 (745 - 1492)	420 (295 - 590)	490 (344 - 689)	560 (393 - 787)	630 (442 - 885)	700 (491 - 984)

Mexico (11 184 468)	136 536 (129 575 - 143 997)	51 877 (49 114 - 54 835)	60 523 (57 300 - 63 974)	69 169 (65 486 - 73 113)	77 815 (73 672 - 82 252)	86 461 (81 857 - 91 392)
Moldova (208 559)	3924 (3148 - 4935)	1733 (1390 - 2179)	2021 (1622 - 2542)	2310 (1854 - 2906)	2599 (2085 - 3269)	2888 (2317 - 3632)
Mongolia (295 899)	8056 (6918 - 9335)	3506 (3011 - 4072)	4090 (3513 - 4751)	4675 (4014 - 5429)	5259 (4516 - 6108)	5844 (5018 - 6787)
Montenegro (37 016)	447 (243 - 838)	197 (107 - 370)	230 (125 - 432)	263 (143 - 493)	296 (161 - 555)	329 (178 - 617)
Morocco (3 146 173)	68 650 (60 752 - 77 146)	27 163 (23 970 - 30 670)	31 691 (27 966 - 35 782)	36 218 (31 961 - 40 894)	40 745 (35 956 - 46 005)	45 272 (39 951 - 51 117)
Mozambique (4 328 490)	176 570 (154 681 - 200 559)	69 865 (61 146 - 79 507)	81 510 (71 337 - 92 759)	93 154 (81 528 - 106 010)	104 798 (91 719 - 119 261)	116 442 (101 910 - 132 512)
Myanmar (4 740 555)	105 792 (59 088 - 187 276)	42 340 (23 645 - 74 860)	49 397 (27 586 - 87 337)	56 454 (31 527 - 99 814)	63 510 (35 468 - 112 291)	70 567 (39 408 - 124 767)
Namibia (303 045)	8577 (6777 - 10 719)	3394 (2678 - 4255)	3959 (3125 - 4964)	4525 (3571 - 5673)	5091 (4017 - 6382)	5656 (4464 - 7091)
Nepal (2 962 654)	64 087 (41 908 - 98 502)	25 649 (16 765 - 39 484)	29 924 (19 559 - 46 065)	34 199 (22 354 - 52 645)	38 473 (25 148 - 59 226)	42 748 (27 942 - 65 807)
New Zealand (306 487)	1639 (1248 - 2170)	772 (588 - 1024)	900 (686 - 1195)	1029 (784 - 1365)	1157 (882 - 1536)	1286 (980 - 1707)
Nicaragua (585 628)	13 579 (10 693 - 17 233)	5159 (4055 - 6566)	6019 (4730 - 7661)	6879 (5406 - 8755)	7739 (6082 - 9850)	8599 (6758 - 10 944)
Niger (3 415 273)	34 620 (21 387 - 56 301)	13 698 (8445 - 22 270)	15 981 (9852 - 25 981)	18 264 (11 260 - 29 693)	20 547 (12 667 - 33 404)	22 831 (14 075 - 37 116)
Nigeria (26 738 733)	877 642 (762 504 - 1 009 298)	347 266 (301 309 - 400 891)	405 144 (351 527 - 467 706)	463 021 (401 745 - 534 521)	520 899 (451 963 - 601 336)	578 777 (502 181 - 668 152)
Norway (309 232)	3194 (2692 - 3802)	1410 (1188 - 1679)	1646 (1386 - 1959)	1881 (1584 - 2239)	2116 (1782 - 2519)	2351 (1980 - 2799)
Oman (325 634)	2296 (1455 - 3470)	999 (634 - 1509)	1166 (740 - 1760)	1332 (846 - 2012)	1499 (951 - 2263)	1665 (1057 - 2515)
Pakistan (21 803 270)	186 422 (151 201 - 229 735)	74 610 (60 397 - 92 089)	87 045 (70 464 - 107 437)	99 480 (80 530 - 122 786)	111 915 (90 596 - 138 134)	124 350 (100 662 - 153 482)
Palestine (626 350)	1432 (638 - 3037)	623 (278 - 1321)	727 (324 - 1541)	831 (370 - 1761)	935 (416 - 1982)	1039 (463 - 2202)
Panama (365 327)	7276 (5616 - 9446)	2765 (2136 - 3592)	3225 (2492 - 4190)	3686 (2848 - 4789)	4147 (3204 - 5388)	4608 (3560 - 5986)
Papua New Guinea (950 656)	27 076 (23 440 - 31 229)	12 743 (10 982 - 14 748)	14 867 (12 812 - 17 207)	16 991 (14 642 - 19 665)	19 115 (16 472 - 22 123)	21 239 (18 303 - 24 581)
Paraguay (654 475)	20 921 (17 092 - 25 650)	7949 (6498 - 9739)	9274 (7581 - 11 363)	10 599 (8664 - 12 986)	11 924 (9747 - 14 609)	13 248 (10 830 - 16 232)
Peru (2 862 578)	77 437 (70 922 - 84 325)	29 422 (26 897 - 32 071)	34 326 (31 380 - 37 416)	39 229 (35 863 - 42 761)	44 133 (40 346 - 48 106)	49 037 (44 829 - 53 452)
Philippines (10 749 238)	178 924 (168 681 - 189 932)	71 609 (67 460 - 76 059)	83 544 (78 703 - 88 735)	95 479 (89 946 - 101 412)	107 414 (101 189 - 114 088)	119 349 (112 433 - 126 765)
Poland (1 887 333)	18 113 (16 811 - 19 527)	7998 (7418 - 8633)	9331 (8655 - 10 072)	10 664 (9891 - 11 510)	11 997 (11 127 - 12 949)	13 330 (12 364 - 14 388)

Portugal (464 522)	3294 (2985 - 3623)	1454 (1318 - 1600)	1697 (1538 - 1867)	1939 (1757 - 2134)	2181 (1977 - 2401)	2424 (2197 - 2667)
Puerto Rico (212 701)	1942 (1683 - 2229)	738 (639 - 847)	861 (745 - 988)	984 (852 - 1129)	1107 (958 - 1270)	1230 (1064 - 1411)
Qatar (120 073)	595 (270 - 1053)	259 (117 - 459)	302 (137 - 536)	345 (156 - 612)	389 (176 - 689)	432 (195 - 766)
Romania (988 253)	21 036 (20 070 - 22 094)	9288 (8846 - 9763)	10 836 (10 320 - 11 390)	12 385 (11 794 - 13 017)	13 933 (13 269 - 14 645)	15 481 (14 743 - 16 272)
Russian Federation (8 138 006)	135 212 (131 924 - 138 545)	59 704 (58 133 - 61 289)	69 654 (67 822 - 71 504)	79 605 (77 511 - 81 718)	89 555 (87 199 - 91 933)	99 506 (96 888 - 102 148)
Rwanda (1 631 965)	59 549 (44 272 - 79 973)	23 562 (17 522 - 31 682)	27 490 (20 442 - 36 962)	31 417 (23 362 - 42 242)	35 344 (26 283 - 47 523)	39 271 (29 203 - 52 803)
Saint Lucia (10 967)	141 (63 - 294)	54 (24 - 112)	63 (28 - 131)	72 (32 - 149)	80 (36 - 168)	89 (40 - 187)
Samoa (23 463)	334 (158 - 678)	157 (74 - 319)	184 (87 - 372)	210 (99 - 426)	236 (112 - 479)	262 (124 - 532)
Sao Tome and Principe (28 676)	794 (200 - 2130)	314 (79 - 842)	366 (92 - 983)	419 (105 - 1123)	471 (118 - 1264)	523 (131 - 1404)
Saudi Arabia (2 664 744)	6421 (3738 - 11 099)	2794 (1624 - 4819)	3260 (1895 - 5622)	3726 (2165 - 6425)	4192 (2436 - 7228)	4657 (2707 - 8031)
Senegal (2 207 619)	96 409 (37 289 - 241 164)	38 147 (14 741 - 95 254)	44 505 (17 197 - 111 130)	50 863 (19 654 - 127 005)	57 221 (22 111 - 142 881)	63 579 (24 568 - 158 756)
Serbia (468 077)	10 148 (9222 - 11 175)	4481 (4073 - 4937)	5228 (4752 - 5760)	5975 (5431 - 6583)	6722 (6110 - 7406)	7468 (6789 - 8228)
Sierra Leone (1 049 663)	18 315 (16 110 - 20 730)	7247 (6358 - 8223)	8454 (7418 - 9594)	9662 (8478 - 10 964)	10 870 (9538 - 12 335)	12 078 (10 597 - 13 705)
Singapore (283 694)	3218 (2108 - 4903)	1288 (844 - 1963)	1503 (985 - 2290)	1717 (1125 - 2617)	1932 (1266 - 2944)	2147 (1407 - 3271)
Slovakia (274 945)	4745 (4338 - 5187)	2095 (1916 - 2292)	2444 (2235 - 2674)	2793 (2555 - 3057)	3143 (2874 - 3439)	3492 (3193 - 3821)
Slovenia (102 069)	705 (467 - 1059)	311 (206 - 466)	363 (240 - 544)	415 (275 - 622)	467 (309 - 700)	519 (343 - 777)
Solomon Islands (76 588)	1745 (1297 - 2304)	821 (608 - 1082)	958 (709 - 1262)	1095 (811 - 1443)	1232 (912 - 1623)	1369 (1013 - 1804)
Somalia (2 203 776)	59 368 (38 611 - 91 443)	23 491 (15 232 - 36 229)	27 406 (17 770 - 42 267)	31 321 (20 309 - 48 305)	35 236 (22 848 - 54 343)	39 151 (25 386 - 60 382)
South Africa (5 408 513)	233 362 (219 697 - 247 286)	92 337 (86 572 - 98 331)	107 726 (101 001 - 114 720)	123 116 (115 429 - 131 108)	138 505 (129 858 - 147 497)	153 894 (144 287 - 163 885)
South Sudan (1 695 917)	49 728 (32 550 - 75 918)	19 676 (12 917 - 30 069)	22 956 (15 070 - 35 081)	26 235 (17 223 - 40 092)	29 515 (19 376 - 45 104)	32 794 (21 529 - 50 115)
Spain (2 272 466)	12 023 (10 837 - 13 330)	5309 (4777 - 5895)	6194 (5574 - 6877)	7079 (6370 - 7860)	7963 (7166 - 8842)	8848 (7962 - 9825)
Sri Lanka (1 676 421)	11 271 (10 393 - 12 207)	4511 (4156 - 4893)	5263 (4848 - 5708)	6015 (5541 - 6524)	6767 (6233 - 7339)	7518 (6926 - 8155)
Sudan (5 360 470)	54 945 (38 132 - 80 092)	21 741 (15 116 - 31 694)	25 364 (17 635 - 36 976)	28 988 (20 154 - 42 259)	32 611 (22 673 - 47 541)	36 235 (25 193 - 52 823)

Suriname (47 779)	1422 (940 - 2097)	540 (356 - 796)	630 (416 - 929)	720 (475 - 1061)	810 (534 - 1194)	900 (594 - 1326)
Swaziland (167 683)	9849 (7879 - 12 191)	3897 (3108 - 4826)	4547 (3626 - 5630)	5196 (4144 - 6434)	5846 (4662 - 7238)	6495 (5180 - 8043)
Sweden (568 431)	4699 (4170 - 5302)	2075 (1839 - 2341)	2420 (2146 - 2731)	2766 (2453 - 3121)	3112 (2759 - 3511)	3458 (3066 - 3901)
Switzerland (407 313)	1671 (1439 - 1927)	738 (636 - 851)	861 (742 - 993)	984 (848 - 1135)	1107 (954 - 1276)	1230 (1060 - 1418)
Syrian Arab Republic (2 217 214)	8701 (4154 - 18 464)	3787 (1809 - 8090)	4418 (2110 - 9438)	5049 (2412 - 10 786)	5680 (2713 - 12 135)	6311 (3015 - 13 483)
Tajikistan (1 009 789)	5965 (4190 - 8453)	2596 (1823 - 3682)	3028 (2127 - 4295)	3461 (2431 - 4909)	3894 (2734 - 5523)	4326 (3038 - 6136)
Tanzania (8 367 875)	573 126 (475 687 - 688 429)	226 775 (187 873 - 272 915)	264 571 (219 185 - 318 401)	302 367 (250 498 - 363 887)	340 162 (281 810 - 409 373)	377 958 (313 122 - 454 858)
Thailand (3 911 333)	57 109 (54 689 - 59 742)	22 856 (21 832 - 23 969)	26 666 (25 471 - 27 964)	30 475 (29 110 - 31 959)	34 285 (32 749 - 35 954)	38 094 (36 387 - 39 949)
The Gambia (307 388)	8630 (4968 - 14 886)	3415 (1969 - 5863)	3984 (2297 - 6840)	4553 (2626 - 7817)	5122 (2954 - 8795)	5691 (3282 - 9772)
The Netherlands (904 567)	4914 (4277 - 5646)	2170 (1887 - 2494)	2531 (2201 - 2910)	2893 (2516 - 3326)	3255 (2830 - 3742)	3616 (3145 - 4157)
Timor-Leste (183 199)	2411 (1345 - 4319)	965 (539 - 1728)	1126 (628 - 2016)	1286 (718 - 2304)	1447 (808 - 2592)	1608 (898 - 2880)
Togo (1 066 033)	26 566 (23 809 - 29 538)	10 512 (9395 - 11 721)	12 264 (10 961 - 13 674)	14 016 (12 527 - 15 628)	15 768 (14 092 - 17 581)	17 520 (15 658 - 19 535)
Trinidad and Tobago (93 918)	1252 (764 - 2047)	476 (290 - 777)	555 (338 - 907)	634 (387 - 1037)	713 (435 - 1166)	793 (483 - 1296)
Tunisia (885 346)	3353 (2684 - 4206)	1327 (1062 - 1666)	1548 (1239 - 1944)	1769 (1416 - 2221)	1990 (1593 - 2499)	2211 (1770 - 2777)
Turkey (6 593 543)	31 841 (28 759 - 35 356)	13 857 (12 478 - 15 422)	16 167 (14 557 - 17 993)	18 476 (16 637 - 20 563)	20 786 (18 717 - 23 133)	23 095 (20 796 - 25 704)
Turkmenistan (565 752)	7983 (5969 - 10 566)	3474 (2591 - 4605)	4053 (3023 - 5373)	4632 (3455 - 6140)	5211 (3887 - 6908)	5790 (4318 - 7675)
Uganda (6 620 397)	378 424 (331 213 - 431 161)	149 735 (130 749 - 171 137)	174 691 (152 540 - 199 660)	199 647 (174 331 - 228 183)	224 603 (196 123 - 256 706)	249 559 (217 914 - 285 229)
Ukraine (2 264 891)	33 354 (31 732 - 35 075)	14 728 (13 979 - 15 502)	17 182 (16 309 - 18 086)	19 637 (18 639 - 20 669)	22 091 (20 969 - 23 253)	24 546 (23 299 - 25 837)
United Arab Emirates (435 202)	4023 (2540 - 5912)	1751 (1104 - 2568)	2042 (1288 - 2996)	2334 (1472 - 3424)	2626 (1656 - 3852)	2918 (1840 - 4280)
United Kingdom (3 853 641)	29 198 (27 556 - 30 943)	12 892 (12 157 - 13 673)	15 041 (14 183 - 15 951)	17 190 (16 209 - 18 230)	19 339 (18 235 - 20 509)	21 487 (20 261 - 22 788)
United States of America (20 177 675)	131 587 (128 403 - 134 848)	57 700 (55 871 - 59 578)	67 317 (65 183 - 69 508)	76 933 (74 495 - 79 437)	86 550 (83 807 - 89 367)	96 167 (93 118 - 99 297)
Uruguay (237 403)	2880 (2285 - 3576)	1094 (870 - 1361)	1277 (1015 - 1588)	1459 (1160 - 1815)	1641 (1305 - 2042)	1824 (1450 - 2268)
Uzbekistan (2 900 550)	30 906 (28 463 - 33 496)	13 450 (12 346 - 14 635)	15 692 (14 403 - 17 074)	17 933 (16 461 - 19 513)	20 175 (18 519 - 21 952)	22 417 (20 576 - 24 391)

Vanuatu<br>(31 810)	509 (278 - 900)	239 (131 - 423)	279 (153 - 494)	319 (174 - 565)	359 (196 - 635)	399 (218 - 706)
Venezuela<br>(2 879 646)	71 413 (65 650 - 77 710)	27 133 (24 903 - 29 564)	31 656 (29 054 - 34 491)	36 178 (33 204 - 39 418)	40 700 (37 355 - 44 345)	45 222 (41 505 - 49 273)
Vietnam<br>(6 992 903)	54 988 (50 414 - 60 134)	22 007 (20 127 - 24 065)	25 675 (23 482 - 28 076)	29 343 (26 836 - 32 087)	33 011 (30 191 - 36 097)	36 679 (33 545 - 40 108)
Yemen<br>(3 640 898)	7781 (5102 - 11 613)	3386 (2213 - 5066)	3950 (2582 - 5910)	4515 (2950 - 6754)	5079 (3319 - 7598)	5644 (3688 - 8443)
Zambia<br>(2 485 403)	181 270 (157 293 - 208 532)	71 725 (62 049 - 82 582)	83 679 (72 390 - 96 345)	95 634 (82 732 - 110 109)	107 588 (93 073 - 123 873)	119 542 (103 415 - 137 636)
Zimbabwe<br>(2 238 806)	157 452 (143 030 - 173 330)	62 301 (56 356 - 68 820)	72 684 (65 748 - 80 290)	83 068 (75 141 - 91 760)	93 451 (84 534 - 103 230)	103 835 (93 926 - 114 700)

Table S3. Country-specific estimated number of lifetime cervical cancer cases expected in the absence of HPV vaccination and preventable through vaccination among women born between 2005 and 2014 by postulated levels of HPV prevalence reduction, attributable to vaccination with a vaccine targeting HPV16/18 and, 31, 33, 45, 52, 58 types.

	Estimated number (95% uncertainty intervals) of cancers					
**Country (Women at risk)**	**Expected in the absence of vaccination**	**Avoided with HPV prevalence reduction, attributable to vaccination**				
		**60%**	**70%**	**80%**	**90%**	**100%**
Afghanistan (4 993 596)	33 107 (23 680 - 46 648)	17 717 (12 666 - 24 907)	20 670 (14 777 - 29 059)	23 623 (16 888 - 33 210)	26 576 (18 999 - 37 361)	29 528 (21 110 - 41 512)
Albania (158 178)	1138 (754 - 1722)	606 (402 - 919)	707 (469 - 1072)	809 (536 - 1225)	910 (603 - 1378)	1011 (670 - 1532)
Algeria (3 940 923)	40 170 (35 139 - 45 656)	20 663 (18 074 - 23 518)	24 107 (21 086 - 27 437)	27 551 (24 099 - 31 357)	30 995 (27 111 - 35 277)	34 439 (30 124 - 39 196)
Angola (4 584 705)	171 867 (130 109 - 226 412)	88 408 (66 898 - 116 620)	103 143 (78 048 - 136 057)	117 878 (89 197 - 155 494)	132 613 (100 347 - 174 931)	147 347 (111 496 - 194 367)
Argentina (3 600 658)	61 460 (55 784 - 67 709)	31 056 (28 179 - 34 244)	36 233 (32 875 - 39 952)	41 409 (37 572 - 45 659)	46 585 (42 268 - 51 366)	51 761 (46 965 - 57 074)
Armenia (184 974)	1875 (1179 - 2983)	1004 (632 - 1595)	1171 (737 - 1860)	1338 (843 - 2126)	1505 (948 - 2392)	1673 (1053 - 2658)
Australia (1 533 702)	8589 (7729 - 9565)	4606 (4130 - 5143)	5374 (4818 - 6000)	6142 (5506 - 6857)	6909 (6195 - 7715)	7677 (6883 - 8572)
Austria (396 884)	2363 (1895 - 2939)	1259 (1010 - 1566)	1469 (1178 - 1827)	1679 (1347 - 2088)	1889 (1515 - 2349)	2099 (1683 - 2610)
Azerbaijan (694 232)	5237 (4574 - 5983)	2802 (2443 - 3203)	3269 (2851 - 3737)	3736 (3258 - 4271)	4204 (3665 - 4804)	4671 (4072 - 5338)
Bahamas (26 573)	306 (163 - 547)	155 (82 - 276)	180 (96 - 322)	206 (110 - 368)	232 (124 - 414)	258 (137 - 460)
Bahrain (96 095)	405 (191 - 760)	217 (103 - 406)	253 (120 - 474)	289 (137 - 542)	325 (154 - 610)	361 (171 - 677)
Bangladesh (15 204 318)	182 334 (130 451 - 255 672)	99 139 (70 901 - 138 764)	115 663 (82 718 - 161 892)	132 186 (94 535 - 185 019)	148 709 (106 352 - 208 146)	165 232 (118 168 - 231 274)
Barbados (17 935)	310 (175 - 529)	157 (88 - 267)	183 (103 - 312)	209 (118 - 357)	235 (133 - 401)	261 (147 - 446)
Belarus (511 896)	6136 (5388 - 6979)	3269 (2870 - 3723)	3814 (3348 - 4344)	4359 (3826 - 4964)	4904 (4305 - 5585)	5449 (4783 - 6205)
Belgium (642 474)	5043 (4480 - 5684)	2687 (2387 - 3028)	3135 (2784 - 3533)	3583 (3182 - 4037)	4030 (3580 - 4542)	4478 (3978 - 5047)
Belize (38 143)	1139 (598 - 2024)	576 (303 - 1023)	672 (353 - 1193)	767 (403 - 1364)	863 (454 - 1534)	959 (504 - 1704)
Benin (1 537 365)	49 686 (41 162 - 59 838)	25 559 (21 192 - 30 782)	29 818 (24 725 - 35 913)	34 078 (28 257 - 41 043)	38 338 (31 789 - 46 173)	42 598 (35 321 - 51 304)
Bhutan (70 699)	1064 (866 - 1287)	579 (471 - 700)	675 (549 - 817)	772 (628 - 933)	868 (706 - 1050)	964 (785 - 1167)

Bolivia (1 137 316)	42 343 (37 683 - 47 631)	21 396 (19 022 - 24 074)	24 962 (22 192 - 28 086)	28 528 (25 362 - 32 098)	32 095 (28 533 - 36 111)	35 661 (31 703 - 40 123)
Bosnia Herzegovina (162 833)	3487 (2971 - 4082)	1858 (1582 - 2174)	2167 (1845 - 2537)	2477 (2109 - 2899)	2786 (2372 - 3262)	3096 (2636 - 3624)
Botswana (235 572)	8551 (7191 - 10 075)	4399 (3694 - 5186)	5132 (4310 - 6050)	5865 (4925 - 6915)	6598 (5541 - 7779)	7331 (6157 - 8643)
Brazil (14 792 741)	185 226 (174 108 - 196 716)	93 597 (87 865 - 99 449)	109 197 (102 510 - 116 024)	124 796 (117 154 - 132 598)	140 396 (131 798 - 149 173)	155 995 (146 442 - 165 748)
Brunei Darussalam (31 490)	615 (382 - 974)	334 (207 - 530)	390 (242 - 618)	446 (276 - 707)	501 (311 - 795)	557 (345 - 883)
Bulgaria (334 066)	6663 (5906 - 7544)	3550 (3144 - 4022)	4142 (3668 - 4692)	4734 (4192 - 5362)	5325 (4716 - 6033)	5917 (5239 - 6703)
Burkina Faso (2 793 581)	122 203 (48 333 - 307 828)	62 861 (24 897 - 158 107)	73 338 (29 046 - 184 458)	83 815 (33 196 - 210 809)	94 292 (37 345 - 237 160)	104 769 (41 495 - 263 511)
Burundi (1 589 898)	95 457 (62 427 - 142 581)	49 103 (32 183 - 73 288)	57 287 (37 547 - 85 503)	65 471 (42 911 - 97 717)	73 655 (48 275 - 109 932)	81 839 (53 639 - 122 147)
Cambodia (1 633 459)	26 186 (14 597 - 46 518)	14 238 (7938 - 25 288)	16 611 (9261 - 29 503)	18 984 (10 584 - 33 717)	21 357 (11 907 - 37 932)	23 730 (13 230 - 42 147)
Cameroon (3 344 918)	110 413 (87 675 - 137 693)	56 796 (45 131 - 70 733)	66 263 (52 653 - 82 521)	75 729 (60 175 - 94 310)	85 195 (67 697 - 106 099)	94 661 (75 218 - 117 888)
Canada (1 946 458)	9374 (8191 - 10 722)	4959 (4333 - 5679)	5785 (5056 - 6625)	6612 (5778 - 7572)	7438 (6500 - 8518)	8265 (7222 - 9465)
Cape Verde (54 412)	1444 (793 - 2537)	743 (407 - 1301)	867 (475 - 1518)	990 (543 - 1735)	1114 (611 - 1952)	1238 (679 - 2169)
Central African Republic (663 673)	14 910 (11 175 - 19 852)	7670 (5744 - 10 225)	8948 (6701 - 11 929)	10 226 (7658 - 13 633)	11 505 (8616 - 15 337)	12 783 (9573 - 17 041)
Chad (2 263 556)	52 520 (39 479 - 69 548)	27 016 (20 321 - 35 775)	31 519 (23 708 - 41 737)	36 022 (27 094 - 47 700)	40 525 (30 481 - 53 662)	45 027 (33 868 - 59 625)
Chile (1 208 357)	12 326 (10 383 - 14 667)	6229 (5245 - 7411)	7267 (6120 - 8646)	8305 (6994 - 9881)	9343 (7868 - 11 116)	10 381 (8742 - 12 351)
China (77 115 987)	867 587 (840 459 - 895 528)	471 728 (456 666 - 487 291)	550 349 (532 777 - 568 506)	628 970 (608 888 - 649 721)	707 591 (684 999 - 730 936)	786 213 (761 110 - 812 151)
Colombia (3 768 405)	55 980 (49 589 - 63 180)	28 287 (25 021 - 31 925)	33 002 (29 191 - 37 246)	37 717 (33 361 - 42 567)	42 431 (37 531 - 47 888)	47 146 (41 701 - 53 209)
Comoros (104 310)	6059 (3903 - 9218)	3117 (2008 - 4753)	3636 (2343 - 5545)	4156 (2678 - 6337)	4675 (3012 - 7129)	5195 (3347 - 7922)
Congo, DR (12 152 010)	319 343 (241 012 - 419 998)	164 270 (123 959 - 216 608)	191 649 (144 619 - 252 709)	219 027 (165 279 - 288 810)	246 405 (185 938 - 324 911)	273 784 (206 598 - 361 013)
Congo (Brazzaville) (730 066)	16 571 (12 993 - 21 138)	8524 (6686 - 10 884)	9945 (7800 - 12 698)	11 365 (8914 - 14 512)	12 786 (10 029 - 16 325)	14 207 (11 143 - 18 139)
Costa Rica (348 535)	4182 (3113 - 5582)	2113 (1574 - 2818)	2465 (1836 - 3288)	2818 (2098 - 3758)	3170 (2360 - 4227)	3522 (2623 - 4697)
Cote d Ivoire (3 344 463)	121 472 (103 648 - 142 403)	62 485 (53 260 - 73 384)	72 900 (62 136 - 85 615)	83 314 (71 013 - 97 845)	93 728 (79 889 - 110 076)	104 142 (88 766 - 122 307)
Croatia (204 038)	1464 (1132 - 1883)	780 (604 - 1003)	910 (704 - 1170)	1040 (805 - 1337)	1170 (905 - 1505)	1300 (1006 - 1672)

Cuba (586 649)	7700 (6662 - 8891)	3891 (3363 - 4493)	4540 (3923 - 5242)	5188 (4484 - 5991)	5837 (5044 - 6740)	6485 (5605 - 7489)
Cyprus (64 836)	357 (235 - 534)	191 (126 - 286)	223 (147 - 333)	255 (168 - 381)	287 (189 - 428)	318 (210 - 476)
Czechia (543 020)	5627 (4918 - 6442)	2998 (2618 - 3432)	3498 (3054 - 4004)	3997 (3491 - 4576)	4497 (3927 - 5148)	4997 (4364 - 5721)
Denmark (313 564)	3345 (2860 - 3908)	1782 (1524 - 2082)	2079 (1778 - 2429)	2376 (2031 - 2776)	2673 (2285 - 3123)	2970 (2539 - 3470)
Djibouti (96 726)	1534 (948 - 2362)	789 (488 - 1216)	920 (569 - 1419)	1052 (650 - 1621)	1183 (731 - 1824)	1315 (813 - 2027)
Dominican Republic (1 032 233)	20 088 (16 448 - 24 491)	10 151 (8309 - 12 380)	11 842 (9694 - 14 444)	13 534 (11 079 - 16 507)	15 226 (12 464 - 18 570)	16 918 (13 849 - 20 634)
Ecuador (1 540 586)	26 933 (24 331 - 29 790)	13 609 (12 290 - 15 050)	15 878 (14 339 - 17 558)	18 146 (16 387 - 20 067)	20 414 (18 436 - 22 575)	22 682 (20 484 - 25 084)
Egypt (10 256 172)	30 537 (22 384 - 41 777)	15 708 (11 527 - 21 458)	18 326 (13 448 - 25 035)	20 944 (15 369 - 28 611)	23 562 (17 290 - 32 187)	26 180 (19 211 - 35 764)
El Salvador (566 651)	11 760 (9766 - 14 125)	5943 (4935 - 7132)	6933 (5758 - 8321)	7923 (6580 - 9510)	8914 (7403 - 10 699)	9904 (8225 - 11 887)
Equatorial Guinea (151 541)	4419 (3195 - 6063)	2273 (1641 - 3123)	2652 (1914 - 3644)	3031 (2188 - 4164)	3409 (2461 - 4685)	3788 (2735 - 5205)
Eritrea (692 925)	10 979 (7134 - 16 771)	5647 (3666 - 8618)	6589 (4277 - 10 055)	7530 (4888 - 11 491)	8471 (5499 - 12 928)	9412 (6110 - 14 364)
Estonia (71 667)	1379 (1103 - 1725)	735 (587 - 920)	857 (685 - 1073)	979 (783 - 1226)	1102 (881 - 1380)	1224 (979 - 1533)
Ethiopia (13 813 867)	298 895 (241 982 - 371 142)	153 752 (124 536 - 191 006)	179 377 (145 292 - 222 840)	205 003 (166 048 - 254 675)	230 628 (186 804 - 286 509)	256 253 (207 560 - 318 343)
Fiji (84 107)	2101 (1707 - 2568)	1127 (911 - 1381)	1315 (1063 - 1611)	1502 (1215 - 1841)	1690 (1367 - 2071)	1878 (1519 - 2301)
Finland (298 748)	1260 (993 - 1607)	671 (529 - 856)	783 (617 - 999)	895 (705 - 1142)	1007 (793 - 1284)	1118 (881 - 1427)
France (3 847 396)	25 943 (23 953 - 28 050)	13 822 (12 761 - 14 951)	16 126 (14 888 - 17 443)	18 430 (17 015 - 19 935)	20 734 (19 142 - 22 426)	23 037 (21 268 - 24 918)
France, Guadeloupe (27 340)	330 (213 - 501)	167 (108 - 253)	195 (126 - 296)	223 (144 - 338)	250 (162 - 380)	278 (180 - 422)
France, La Reunion (67 334)	728 (540 - 968)	374 (278 - 498)	437 (324 - 581)	499 (370 - 664)	562 (417 - 747)	624 (463 - 830)
France, Martinique (23 264)	200 (117 - 316)	101 (59 - 160)	118 (69 - 186)	135 (79 - 213)	152 (89 - 239)	169 (99 - 266)
France, New Caledonia (19 996)	348 (206 - 569)	187 (110 - 306)	218 (129 - 357)	249 (147 - 408)	280 (166 - 459)	311 (184 - 510)
French Guyana (30 677)	738 (373 - 1365)	373 (188 - 691)	435 (219 - 806)	497 (251 - 921)	559 (282 - 1036)	622 (313 - 1151)
French Polynesia (21 264)	262 (133 - 484)	141 (71 - 259)	164 (83 - 302)	187 (95 - 346)	211 (107 - 389)	234 (119 - 432)
Gabon (236 188)	5594 (4351 - 7049)	2878 (2235 - 3632)	3357 (2607 - 4237)	3837 (2979 - 4842)	4317 (3352 - 5447)	4796 (3724 - 6053)

Georgia<br>(239 957)	2543 (1709 - 3770)	1361 (914 - 2012)	1588 (1066 - 2348)	1815 (1219 - 2683)	2041 (1371 - 3018)	2268 (1523 - 3354)
Germany<br>(3 476 839)	26 017 (24 445 - 27 687)	13 861 (13 015 - 14 761)	16 172 (15 184 - 17 221)	18 482 (17 353 - 19 682)	20 792 (19 522 - 22 142)	23 102 (21 691 - 24 602)
Ghana<br>(3 574 916)	126 482 (112 467 - 141 862)	65 063 (57 865 - 73 015)	75 906 (67 509 - 85 184)	86 750 (77 154 - 97 354)	97 594 (86 798 - 109 523)	108 438 (96 442 - 121 692)
Greece<br>(531 559)	4165 (3152 - 5478)	2219 (1677 - 2919)	2589 (1956 - 3406)	2959 (2235 - 3892)	3329 (2515 - 4379)	3699 (2794 - 4865)
Guam<br>(13 054)	251 (193 - 321)	135 (103 - 172)	157 (121 - 201)	179 (138 - 230)	202 (155 - 259)	224 (172 - 287)
Guatemala<br>(1 913 663)	42 917 (37 066 - 49 600)	21 687 (18 722 - 25 048)	25 301 (21 843 - 29 223)	28 915 (24 963 - 33 397)	32 530 (28 084 - 37 572)	36 144 (31 204 - 41 747)
Guinea-Bissau<br>(251 969)	10 682 (4160 - 27 164)	5495 (2144 - 14 031)	6411 (2501 - 16 369)	7327 (2858 - 18 708)	8242 (3216 - 21 046)	9158 (3573 - 23 385)
Guinea<br>(1 749 245)	99 417 (79 879 - 123 716)	51 140 (41 051 - 63 597)	59 664 (47 892 - 74 197)	68 187 (54 734 - 84 796)	76 710 (61 576 - 95 396)	85 234 (68 418 - 105 995)
Guyana<br>(72 043)	2638 (2042 - 3366)	1333 (1031 - 1700)	1555 (1203 - 1984)	1777 (1375 - 2267)	1999 (1547 - 2550)	2221 (1719 - 2834)
Haiti<br>(1 182 320)	24 616 (7602 - 75 534)	12 439 (3830 - 38 006)	14 512 (4469 - 44 341)	16 585 (5107 - 50 675)	18 658 (5746 - 57 010)	20 731 (6384 - 63 344)
Honduras<br>(959 120)	20 994 (17 217 - 25 458)	10 608 (8698 - 12 848)	12 376 (10 148 - 14 990)	14 144 (11 598 - 17 131)	15 913 (13 048 - 19 273)	17 681 (14 497 - 21 414)
Hungary<br>(458 141)	7927 (6812 - 9268)	4224 (3629 - 4939)	4928 (4234 - 5762)	5632 (4838 - 6585)	6336 (5443 - 7408)	7040 (6048 - 8231)
Iceland<br>(22 234)	130 (74 - 224)	69 (39 - 119)	81 (46 - 139)	92 (52 - 159)	104 (59 - 179)	115 (65 - 199)
India<br>(117 883 612)	1 803 757 (1 738 203 - 1 873 304)	999 728 (954 766 - 1 046 504)	1 166 350 (1 113 893 - 1 220 922)	1 332 971 (1 273 021 - 1 395 339)	1 499 593 (1 432 148 - 1 569 757)	1 666 214 (1 591 276 - 1 744 174)
Indonesia<br>(23 396 629)	556 851 (515 003 - 602 033)	302 773 (280 103 - 327 419)	353 235 (326 787 - 381 989)	403 697 (373 471 - 436 559)	454 159 (420 155 - 491 128)	504 621 (466 839 - 545 698)
Iran, Islamic Republic of<br>(6 365 497)	17 005 (15 213 - 18 965)	9100 (8134 - 10 157)	10 617 (9490 - 11 849)	12 134 (10 846 - 13 542)	13 651 (12 201 - 15 235)	15 167 (13 557 - 16 928)
Iraq<br>(4 936 719)	10 341 (8428 - 12 585)	5534 (4506 - 6741)	6456 (5257 - 7865)	7379 (6008 - 8988)	8301 (6759 - 10 112)	9223 (7510 - 11 235)
Ireland<br>(340 925)	3650 (2825 - 4726)	1945 (1506 - 2520)	2269 (1757 - 2940)	2593 (2009 - 3360)	2917 (2260 - 3779)	3241 (2511 - 4199)
Israel<br>(747 276)	3972 (3200 - 4912)	2126 (1709 - 2634)	2480 (1993 - 3074)	2834 (2278 - 3513)	3188 (2563 - 3952)	3543 (2848 - 4391)
Italy<br>(2 644 557)	17 468 (16 331 - 18 694)	9307 (8698 - 9959)	10 858 (10 148 - 11 619)	12 409 (11 597 - 13 279)	13 961 (13 047 - 14 938)	15 512 (14 497 - 16 598)
Jamaica<br>(213 952)	6547 (4355 - 9782)	3308 (2201 - 4946)	3859 (2567 - 5770)	4411 (2934 - 6595)	4962 (3301 - 7419)	5513 (3668 - 8243)
Japan<br>(5 360 201)	71 887 (68 512 - 75 442)	39 087 (37 238 - 41 035)	45 601 (43 445 - 47 875)	52 116 (49 651 - 54 714)	58 630 (55 857 - 61 553)	65 145 (62 064 - 68 392)
Jordan<br>(1 122 839)	4069 (2843 - 5721)	2177 (1521 - 3064)	2540 (1774 - 3575)	2903 (2027 - 4085)	3266 (2281 - 4596)	3629 (2534 - 5106)

Kazakhstan (1 651 384)	27 500 (25 911 - 29 210)	14 717 (13 844 - 15 656)	17 169 (16 151 - 18 265)	19 622 (18 458 - 20 874)	22 075 (20 766 - 23 484)	24 528 (23 073 - 26 093)
Kenya (6 630 383)	274 125 (241 202 - 310 540)	141 010 (123 871 - 159 922)	164 512 (144 516 - 186 575)	188 013 (165 161 - 213 229)	211 515 (185 806 - 239 883)	235 017 (206 451 - 266 536)
Korea, North (1 697 972)	15 751 (13 542 - 18 342)	8564 (7368 - 9962)	9991 (8596 - 11 623)	11 419 (9824 - 13 283)	12 846 (11 052 - 14 943)	14 273 (12 280 - 16 604)
Korea, South (2 206 345)	18 752 (17 696 - 19 837)	10 196 (9616 - 10 788)	11 895 (11 219 - 12 586)	13 594 (12 822 - 14 384)	15 294 (14 425 - 16 182)	16 993 (16 027 - 17 980)
Kuwait (280 477)	1294 (732 - 2086)	693 (392 - 1115)	808 (457 - 1300)	923 (522 - 1486)	1039 (587 - 1672)	1154 (653 - 1858)
Kyrgyzstan (619 807)	13 002 (9793 - 17 291)	6958 (5244 - 9269)	8118 (6117 - 10 814)	9277 (6991 - 12 358)	10 437 (7865 - 13 903)	11 596 (8739 - 15 448)
Laos (737 729)	9693 (5400 - 17 541)	5271 (2941 - 9525)	6149 (3431 - 11 113)	7027 (3922 - 12 700)	7906 (4412 - 14 288)	8784 (4902 - 15 875)
Latvia (99 928)	2354 (1803 - 3054)	1254 (961 - 1628)	1463 (1121 - 1900)	1672 (1281 - 2171)	1881 (1442 - 2442)	2090 (1602 - 2714)
Lebanon (449 624)	2503 (1979 - 3136)	1340 (1059 - 1677)	1563 (1235 - 1957)	1786 (1412 - 2236)	2009 (1588 - 2516)	2233 (1764 - 2795)
Lesotho (257 428)	13 973 (10 653 - 18 170)	7188 (5483 - 9349)	8386 (6396 - 10 907)	9584 (7310 - 12 465)	10 782 (8224 - 14 023)	11 980 (9138 - 15 581)
Liberia (636 436)	29 393 (11 400 - 74 472)	15 120 (5911 - 38 201)	17 640 (6896 - 44 567)	20 159 (7881 - 50 934)	22 679 (8866 - 57 301)	25 199 (9851 - 63 668)
Libya (585 328)	8096 (5625 - 11 596)	4165 (2899 - 5967)	4859 (3383 - 6961)	5553 (3866 - 7956)	6247 (4349 - 8950)	6941 (4832 - 9945)
Lithuania (138 389)	2521 (2159 - 2950)	1343 (1150 - 1571)	1567 (1341 - 1833)	1791 (1533 - 2095)	2015 (1725 - 2357)	2239 (1916 - 2619)
Luxembourg (31 122)	193 (83 - 431)	103 (44 - 230)	120 (52 - 268)	137 (59 - 307)	154 (67 - 345)	171 (74 - 383)
Macedonia (111 363)	1223 (872 - 1734)	652 (465 - 926)	760 (542 - 1080)	869 (619 - 1234)	978 (697 - 1388)	1086 (774 - 1543)
Madagascar (3 417 425)	152 716 (100 781 - 229 850)	78 557 (51 824 - 118 141)	91 650 (60 461 - 137 831)	104 743 (69 099 - 157 521)	117 836 (77 736 - 177 211)	130 929 (86 373 - 196 901)
Malawi (2 697 638)	204 058 (172 599 - 239 887)	104 967 (88 721 - 123 524)	122 462 (103 508 - 144 112)	139 957 (118 295 - 164 699)	157 451 (133 081 - 185 286)	174 946 (147 868 - 205 874)
Malaysia (2 461 366)	27 359 (25 156 - 29 687)	14 876 (13 663 - 16 157)	17 355 (15 940 - 18 850)	19 834 (18 217 - 21 542)	22 314 (20 495 - 24 235)	24 793 (22 772 - 26 928)
Maldives (32 519)	843 (558 - 1233)	458 (303 - 670)	535 (353 - 782)	611 (404 - 893)	688 (454 - 1005)	764 (505 - 1116)
Mali (2 848 972)	146 618 (127 997 - 167 492)	75 421 (65 825 - 86 384)	87 991 (76 795 - 100 781)	100 561 (87 766 - 115 179)	113 131 (98 737 - 129 576)	125 701 (109 708 - 143 974)
Malta (19 795)	68 (34 - 134)	36 (18 - 71)	43 (21 - 83)	49 (24 - 95)	55 (27 - 107)	61 (30 - 119)
Mauritania (569 053)	20 975 (8133 - 53 704)	10 790 (4177 - 27 653)	12 588 (4873 - 32 261)	14 386 (5569 - 36 870)	16 185 (6265 - 41 479)	17 983 (6961 - 46 088)
Mauritius (76 281)	1061 (745 - 1492)	546 (383 - 768)	637 (447 - 896)	728 (511 - 1024)	819 (575 - 1152)	910 (639 - 1280)

Mexico (11 184 468)	136 536 (129 575 - 143 997)	68 993 (65 448 - 72 775)	80 492 (76 356 - 84 904)	91 991 (87 264 - 97 033)	103 490 (98 172 - 109 162)	114 989 (109 080 - 121 291)
Moldova (208 559)	3924 (3148 - 4935)	2091 (1678 - 2628)	2439 (1958 - 3066)	2787 (2238 - 3504)	3136 (2517 - 3942)	3484 (2797 - 4380)
Mongolia (295 899)	8056 (6918 - 9335)	4311 (3699 - 4996)	5030 (4315 - 5829)	5749 (4932 - 6661)	6467 (5548 - 7494)	7186 (6164 - 8327)
Montenegro (37 016)	447 (243 - 838)	238 (129 - 446)	278 (151 - 521)	317 (172 - 595)	357 (194 - 670)	397 (215 - 744)
Morocco (3 146 173)	68 650 (60 752 - 77 146)	35 313 (31 227 - 39 670)	41 199 (36 432 - 46 282)	47 085 (41 636 - 52 894)	52 970 (46 841 - 59 505)	58 856 (52 045 - 66 117)
Mozambique (4 328 490)	176 570 (154 681 - 200 559)	90 828 (79 690 - 103 283)	105 966 (92 971 - 120 497)	121 104 (106 253 - 137 710)	136 242 (119 534 - 154 924)	151 380 (132 816 - 172 138)
Myanmar (4 740 555)	105 792 (59 088 - 187 276)	57 522 (32 135 - 101 840)	67 109 (37 491 - 118 814)	76 695 (42 846 - 135 787)	86 282 (48 202 - 152 761)	95 869 (53 558 - 169 734)
Namibia (303 045)	8577 (6777 - 10 719)	4412 (3486 - 5512)	5147 (4067 - 6430)	5883 (4648 - 7349)	6618 (5229 - 8268)	7353 (5810 - 9186)
Nepal (2 962 654)	64 087 (41 908 - 98 502)	34 845 (22 766 - 53 570)	40 653 (26 561 - 62 499)	46 461 (30 355 - 71 427)	52 268 (34 150 - 80 355)	58 076 (37 944 - 89 284)
New Zealand (306 487)	1639 (1248 - 2170)	879 (670 - 1165)	1026 (781 - 1359)	1172 (893 - 1553)	1319 (1005 - 1747)	1465 (1116 - 1941)
Nicaragua (585 628)	13 579 (10 693 - 17 233)	6862 (5404 - 8715)	8005 (6305 - 10 167)	9149 (7205 - 11 620)	10 293 (8106 - 13 072)	11 436 (9007 - 14 525)
Niger (3 415 273)	34 620 (21 387 - 56 301)	17 808 (11 006 - 29 006)	20 776 (12 840 - 33 841)	23 744 (14 675 - 38 675)	26 713 (16 509 - 43 509)	29 681 (18 343 - 48 344)
Nigeria (26 738 733)	877 642 (762 504 - 1 009 298)	451 459 (392 221 - 519 019)	526 703 (457 591 - 605 523)	601 946 (522 962 - 692 026)	677 189 (588 332 - 778 529)	752 432 (653 702 - 865 032)
Norway (309 232)	3194 (2692 - 3802)	1702 (1434 - 2026)	1986 (1673 - 2364)	2269 (1912 - 2701)	2553 (2151 - 3039)	2837 (2390 - 3376)
Oman (325 634)	2296 (1455 - 3470)	1229 (779 - 1854)	1434 (909 - 2163)	1638 (1039 - 2472)	1843 (1169 - 2781)	2048 (1299 - 3090)
Pakistan (21 803 270)	186 422 (151 201 - 229 735)	101 362 (82 262 - 124 905)	118 256 (95 973 - 145 722)	135 149 (109 683 - 166 540)	152 043 (123 393 - 187 357)	168 937 (137 104 - 208 175)
Palestine (626 350)	1432 (638 - 3037)	766 (341 - 1627)	894 (398 - 1898)	1022 (455 - 2169)	1149 (512 - 2440)	1277 (569 - 2711)
Panama (365 327)	7276 (5616 - 9446)	3677 (2837 - 4777)	4290 (3310 - 5574)	4902 (3783 - 6370)	5515 (4256 - 7166)	6128 (4729 - 7962)
Papua New Guinea (950 656)	27 076 (23 440 - 31 229)	14 520 (12 543 - 16 786)	16 941 (14 633 - 19 584)	19 361 (16 724 - 22 381)	21 781 (18 814 - 25 179)	24 201 (20 904 - 27 977)
Paraguay (654 475)	20 921 (17 092 - 25 650)	10 572 (8642 - 12 955)	12 334 (10 083 - 15 114)	14 096 (11 523 - 17 273)	15 858 (12 963 - 19 432)	17 620 (14 404 - 21 591)
Peru (2 862 578)	77 437 (70 922 - 84 325)	39 130 (35 822 - 42 634)	45 651 (41 792 - 49 739)	52 173 (47 762 - 56 845)	58 695 (53 732 - 63 951)	65 216 (59 703 - 71 056)
Philippines (10 749 238)	178 924 (168 681 - 189 932)	97 285 (91 725 - 103 283)	113 499 (107 013 - 120 497)	129 714 (122 300 - 137 711)	145 928 (137 588 - 154 924)	162 142 (152 875 - 172 138)
Poland (1 887 333)	18 113 (16 811 - 19 527)	9650 (8952 - 10 406)	11 259 (10 444 - 12 140)	12 867 (11 936 - 13 875)	14 476 (13 428 - 15 609)	16 084 (14 920 - 17 343)

Portugal (464 522)	3294 (2985 - 3623)	1755 (1590 - 1931)	2047 (1855 - 2253)	2340 (2120 - 2575)	2632 (2385 - 2897)	2925 (2650 - 3219)
Puerto Rico (212 701)	1942 (1683 - 2229)	981 (850 - 1126)	1145 (992 - 1314)	1308 (1133 - 1502)	1472 (1275 - 1689)	1635 (1416 - 1877)
Qatar (120 073)	595 (270 - 1053)	319 (144 - 566)	372 (168 - 661)	425 (192 - 755)	478 (216 - 850)	531 (241 - 944)
Romania (988 253)	21 036 (20 070 - 22 094)	11 208 (10 685 - 11 776)	13 076 (12 466 - 13 739)	14 944 (14 247 - 15 701)	16 812 (16 028 - 17 664)	18 679 (17 809 - 19 627)
Russian Federation (8 138 006)	135 212 (131 924 - 138 545)	72 040 (70 248 - 73 878)	84 047 (81 956 - 86 191)	96 054 (93 664 - 98 504)	108 060 (105 372 - 110 817)	120 067 (117 080 - 123 129)
Rwanda (1 631 965)	59 549 (44 272 - 79 973)	30 632 (22 734 - 41 154)	35 738 (26 523 - 48 013)	40 843 (30 312 - 54 872)	45 948 (34 101 - 61 731)	51 054 (37 890 - 68 590)
Saint Lucia (10 967)	141 (63 - 294)	71 (32 - 149)	83 (37 - 174)	95 (43 - 198)	107 (48 - 223)	119 (53 - 248)
Samoa (23 463)	334 (158 - 678)	179 (85 - 364)	209 (99 - 424)	239 (113 - 485)	269 (127 - 545)	299 (141 - 606)
Sao Tome and Principe (28 676)	794 (200 - 2130)	408 (103 - 1095)	476 (120 - 1277)	544 (137 - 1460)	612 (154 - 1642)	680 (171 - 1825)
Saudi Arabia (2 664 744)	6421 (3738 - 11 099)	3436 (1995 - 5940)	4009 (2328 - 6930)	4582 (2661 - 7920)	5154 (2993 - 8910)	5727 (3326 - 9900)
Senegal (2 207 619)	96 409 (37 289 - 241 164)	49 593 (19 176 - 123 974)	57 858 (22 372 - 144 636)	66 124 (25 569 - 165 298)	74 389 (28 765 - 185 960)	82 655 (31 961 - 206 623)
Serbia (468 077)	10 148 (9222 - 11 175)	5407 (4915 - 5953)	6308 (5735 - 6946)	7209 (6554 - 7938)	8110 (7373 - 8930)	9012 (8192 - 9922)
Sierra Leone (1 049 663)	18 315 (16 110 - 20 730)	9421 (8270 - 10 666)	10 991 (9648 - 12 443)	12 561 (11 027 - 14 221)	14 131 (12 405 - 15 998)	15 702 (13 783 - 17 776)
Singapore (283 694)	3218 (2108 - 4903)	1750 (1146 - 2665)	2041 (1336 - 3109)	2333 (1527 - 3553)	2625 (1718 - 3997)	2916 (1909 - 4442)
Slovakia (274 945)	4745 (4338 - 5187)	2528 (2312 - 2765)	2949 (2698 - 3225)	3371 (3083 - 3686)	3792 (3468 - 4147)	4213 (3854 - 4608)
Slovenia (102 069)	705 (467 - 1059)	376 (249 - 564)	438 (290 - 658)	501 (331 - 751)	564 (373 - 845)	626 (414 - 939)
Solomon Islands (76 588)	1745 (1297 - 2304)	936 (696 - 1236)	1092 (812 - 1441)	1248 (927 - 1647)	1404 (1043 - 1853)	1560 (1159 - 2059)
Somalia (2 203 776)	59 368 (38 611 - 91 443)	30 539 (19 828 - 47 038)	35 629 (23 133 - 54 878)	40 719 (26 438 - 62 718)	45 809 (29 742 - 70 557)	50 898 (33 047 - 78 397)
South Africa (5 408 513)	233 362 (219 697 - 247 286)	120 041 (112 889 - 127 402)	140 048 (131 704 - 148 635)	160 055 (150 519 - 169 869)	180 062 (169 334 - 191 102)	200 069 (188 149 - 212 336)
South Sudan (1 695 917)	49 728 (32 550 - 75 918)	25 580 (16 802 - 39 073)	29 843 (19 603 - 45 585)	34 107 (22 403 - 52 097)	38 370 (25 203 - 58 609)	42 633 (28 004 - 65 122)
Spain (2 272 466)	12 023 (10 837 - 13 330)	6406 (5769 - 7107)	7474 (6730 - 8292)	8541 (7692 - 9477)	9609 (8653 - 10 661)	10 677 (9615 - 11 846)
Sri Lanka (1 676 421)	11 271 (10 393 - 12 207)	6128 (5652 - 6638)	7150 (6594 - 7745)	8171 (7536 - 8851)	9193 (8478 - 9957)	10 214 (9420 - 11 064)
Sudan (5 360 470)	54 945 (38 132 - 80 092)	28 264 (19 623 - 41 205)	32 975 (22 893 - 48 073)	37 685 (26 164 - 54 940)	42 396 (29 434 - 61 808)	47 107 (32 704 - 68 675)

Suriname (47 779)	1422 (940 - 2097)	718 (475 - 1060)	838 (554 - 1237)	958 (633 - 1413)	1078 (712 - 1590)	1197 (791 - 1767)
Swaziland (167 683)	9849 (7879 - 12 191)	5066 (4047 - 6279)	5911 (4722 - 7325)	6755 (5397 - 8372)	7599 (6071 - 9418)	8444 (6746 - 10 465)
Sweden (568 431)	4699 (4170 - 5302)	2503 (2222 - 2824)	2921 (2592 - 3294)	3338 (2963 - 3765)	3755 (3333 - 4236)	4172 (3703 - 4706)
Switzerland (407 313)	1671 (1439 - 1927)	890 (768 - 1027)	1039 (896 - 1198)	1187 (1024 - 1369)	1335 (1152 - 1540)	1484 (1280 - 1711)
Syrian Arab Republic (2 217 214)	8701 (4154 - 18 464)	4656 (2224 - 9892)	5432 (2595 - 11 540)	6209 (2966 - 13 189)	6985 (3336 - 14 837)	7761 (3707 - 16 486)
Tajikistan (1 009 789)	5965 (4190 - 8453)	3192 (2242 - 4524)	3724 (2616 - 5278)	4256 (2989 - 6032)	4788 (3363 - 6786)	5320 (3737 - 7539)
Tanzania (8 367 875)	573 126 (475 687 - 688 429)	294 816 (244 163 - 354 855)	343 952 (284 857 - 413 998)	393 088 (325 551 - 473 141)	442 225 (366 244 - 532 283)	491 361 (406 938 - 591 426)
Thailand (3 911 333)	57 109 (54 689 - 59 742)	31 052 (29 742 - 32 488)	36 227 (34 699 - 37 902)	41 402 (39 656 - 43 317)	46 578 (44 613 - 48 731)	51 753 (49 570 - 54 146)
The Gambia (307 388)	8630 (4968 - 14 886)	4439 (2563 - 7651)	5179 (2990 - 8927)	5919 (3417 - 10 202)	6659 (3845 - 11 477)	7399 (4272 - 12 752)
The Netherlands (904 567)	4914 (4277 - 5646)	2618 (2279 - 3010)	3054 (2659 - 3512)	3491 (3039 - 4014)	3927 (3419 - 4516)	4363 (3799 - 5017)
Timor-Leste (183 199)	2411 (1345 - 4319)	1311 (731 - 2346)	1529 (852 - 2737)	1748 (974 - 3128)	1966 (1096 - 3519)	2185 (1218 - 3910)
Togo (1 066 033)	26 566 (23 809 - 29 538)	13 666 (12 232 - 15 217)	15 943 (14 271 - 17 753)	18 221 (16 310 - 20 289)	20 499 (18 348 - 22 825)	22 776 (20 387 - 25 361)
Trinidad and Tobago (93 918)	1252 (764 - 2047)	633 (385 - 1037)	738 (450 - 1210)	843 (514 - 1382)	949 (578 - 1555)	1054 (642 - 1728)
Tunisia (885 346)	3353 (2684 - 4206)	1725 (1381 - 2167)	2012 (1611 - 2528)	2300 (1842 - 2889)	2587 (2072 - 3250)	2875 (2302 - 3611)
Turkey (6 593 543)	31 841 (28 759 - 35 356)	17 040 (15 378 - 18 953)	19 880 (17 941 - 22 111)	22 720 (20 504 - 25 270)	25 560 (23 067 - 28 429)	28 400 (25 630 - 31 588)
Turkmenistan (565 752)	7983 (5969 - 10 566)	4272 (3187 - 5660)	4984 (3718 - 6603)	5696 (4249 - 7546)	6408 (4780 - 8489)	7120 (5311 - 9433)
Uganda (6 620 397)	378 424 (331 213 - 431 161)	194 662 (170 198 - 221 972)	227 105 (198 565 - 258 968)	259 549 (226 931 - 295 963)	291 992 (255 297 - 332 959)	324 436 (283 664 - 369 954)
Ukraine (2 264 891)	33 354 (31 732 - 35 075)	17 771 (16 893 - 18 688)	20 733 (19 708 - 21 802)	23 694 (22 523 - 24 917)	26 656 (25 339 - 28 031)	29 618 (28 154 - 31 146)
United Arab Emirates (435 202)	4023 (2540 - 5912)	2153 (1361 - 3167)	2512 (1588 - 3695)	2870 (1814 - 4223)	3229 (2041 - 4751)	3588 (2268 - 5278)
United Kingdom (3 853 641)	29 198 (27 556 - 30 943)	15 556 (14 688 - 16 495)	18 149 (17 136 - 19 244)	20 742 (19 584 - 21 993)	23 335 (22 033 - 24 742)	25 927 (24 481 - 27 491)
United States of America (20 177 675)	131 587 (128 403 - 134 848)	69 608 (67 704 - 71 586)	81 209 (78 988 - 83 518)	92 811 (90 272 - 95 449)	104 412 (101 555 - 107 380)	116 013 (112 839 - 119 311)
Uruguay (237 403)	2880 (2285 - 3576)	1455 (1156 - 1806)	1698 (1349 - 2107)	1940 (1542 - 2408)	2183 (1735 - 2709)	2425 (1927 - 3010)
Uzbekistan (2 900 550)	30 906 (28 463 - 33 496)	16 539 (15 217 - 17 926)	19 296 (17 753 - 20 913)	22 052 (20 289 - 23 901)	24 809 (22 825 - 26 888)	27 565 (25 361 - 29 876)

Vanuatu (31 810)	509 (278 - 900)	273 (149 - 484)	318 (174 - 564)	364 (199 - 645)	409 (223 - 725)	455 (248 - 806)
Venezuela (2 879 646)	71 413 (65 650 - 77 710)	36 086 (33 174 - 39 297)	42 100 (38 704 - 45 846)	48 115 (44 233 - 52 396)	54 129 (49 762 - 58 945)	60 143 (55 291 - 65 495)
Vietnam (6 992 903)	54 988 (50 414 - 60 134)	29 898 (27 416 - 32 686)	34 881 (31 986 - 38 134)	39 864 (36 555 - 43 582)	44 847 (41 125 - 49 029)	49 830 (45 694 - 54 477)
Yemen (3 640 898)	7781 (5102 - 11 613)	4164 (2733 - 6220)	4858 (3188 - 7256)	5552 (3644 - 8293)	6246 (4099 - 9330)	6940 (4555 - 10 366)
Zambia (2 485 403)	181 270 (157 293 - 208 532)	93 246 (80 784 - 107 314)	108 787 (94 248 - 125 200)	124 328 (107 713 - 143 085)	139 868 (121 177 - 160 971)	155 409 (134 641 - 178 857)
Zimbabwe (2 238 806)	157 452 (143 030 - 173 330)	80 994 (73 383 - 89 274)	94 493 (85 614 - 104 153)	107 992 (97 844 - 119 032)	121 490 (110 075 - 133 912)	134 989 (122 305 - 148 791)

Supplementary Figure 1. Country-specific ratio of the future number of cervical cancers cases expected in the cohort born in year 2005 in the absence of vaccination to the number of cases estimated by GLOBOCAN for year 2018 by human development index. Correlation factor = -0·5

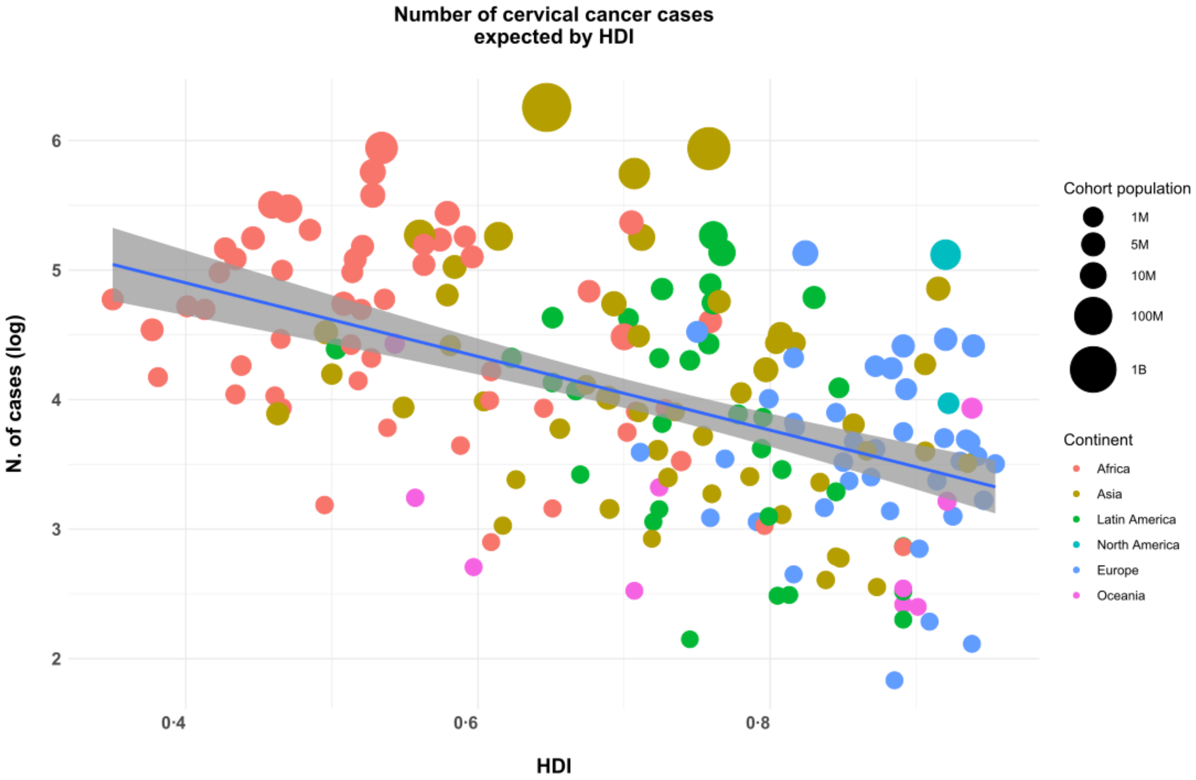

Supplement: Supplementary appendix [file mmc1.pdf]
